# Biocompatible Chemistry: A Plug‐and‐Play Toolbox for Chemical Biology Research

**DOI:** 10.1002/cbic.202500590

**Published:** 2025-09-26

**Authors:** Adam R. Lovato, Zeng Lin, Qingfei Zheng

**Affiliations:** ^1^ Department of Medicinal Chemistry and Molecular Pharmacology College of Pharmacy Purdue University West Lafayette IN 47907 USA; ^2^ Institute for Cancer Research Purdue University West Lafayette IN 47907 USA

**Keywords:** biocompatible chemistry, biomacromolecule labeling, biomarker validation, bioorthogonal chemistry, click chemistry, drug discovery

## Abstract

Chemistry is referred to as the central science that has been widely applied in biological, material, and clinical research. Specifically, chemical biology is an interdisciplinary subject, where well‐designed chemical tools and approaches are employed to solve complex biological questions. In the past few decades, the development of biocompatible reactions, which include click chemistry and bioorthogonal chemistry, is one of the most significant advances in the chemical biology field. In short, biocompatible chemistry enables the cleavage and formation of chemical bonds under physiological conditions, which can be thus utilized for specific labeling of cellular biomacromolecules of interest, biomarker/target validation, signaling pathway identification, drug discovery, and so on. The plug‐and‐play nature of biocompatible chemistry makes it a powerful toolbox for investigating complicated biological systems and producing drug leads for clinical usages. In this article, the commonly used biocompatible reactions and the recent advances of their representative applications in chemical biology research is summarized, thus highlighting the diverse potential of biocompatible chemistry as a plug‐and‐play toolbox for future basic and translational studies.

## Graphical abstract

The “plug‐and‐play” nature of biocompatible chemistry makes it a powerful toolbox for investigating complicated biological systems and producing drug leads for clinical usages. This review summarizes the commonly used biocompatible reactions and the recent advances of their representative applications in chemical biology research, thus highlighting the diverse potential of biocompatible chemistry as a “plug‐and‐play” toolbox for future basic and translational studies.

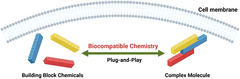

## 1 Introduction

Modern chemical biology and its applications in research experienced a renaissance in the late 1990s and early 2000s driven by the abstract ideas and discoveries of Nobel laureates, Carolyn R. Bertozzi, Morten Meldal, and K. Barry Sharpless. At a conference in 1999, Sharpless proposed that synthesis of compounds should follow a more streamlined approach using a limited set of reactions that are highly robust and efficient.^[^ ^1^ ^]^ Sharpless et al. would further elaborate on this concept in 2001, defining the idea of ‘click chemistry’ as a set of ‘spring‐loaded’ reactions that could synthesize compounds like connecting building blocks.^[^ ^2^ ^]^

Later that same year Sharpless^[^ ^3^ ^]^ and Meldal^[^ ^4^ ^]^ would independently discover that in the presence of a Cu(I) catalyst, organoazides and terminal alkynes can react in a regioselective manner to create a 1,2,3‐triazole. This reaction was given the name copper‐catalyzed azide–alkyne cycloaddition (CuAAC) and remains the most recognizable and archetypal click‐type reactions. The idea of simple, high yielding reactions would change how chemists viewed synthetic challenges, and moreover, how chemical synthesis would be performed within a living organism.

At the same time, Bertozzi was adapting chemical reactions to be used within living cells; a theory that she would later name ‘bioorthogonal chemistry’. In 2000, Bertozzi would adapt the Staudinger reaction, now called Staudinger ligation, to label azidosugars on cell surfaces. Initially discovered in the early 1900s, the reaction utilizes a nucleophilic phosphane and an azide handle to yield an iminophosphorane.^[^ ^5^ ^]^ Using a relatively simple cell labeling model, Bertozzi was the first to show that chemical synthesis could be performed in living systems and the Staudinger ligation became the first bioorthogonal reaction.

**Figure 1:**
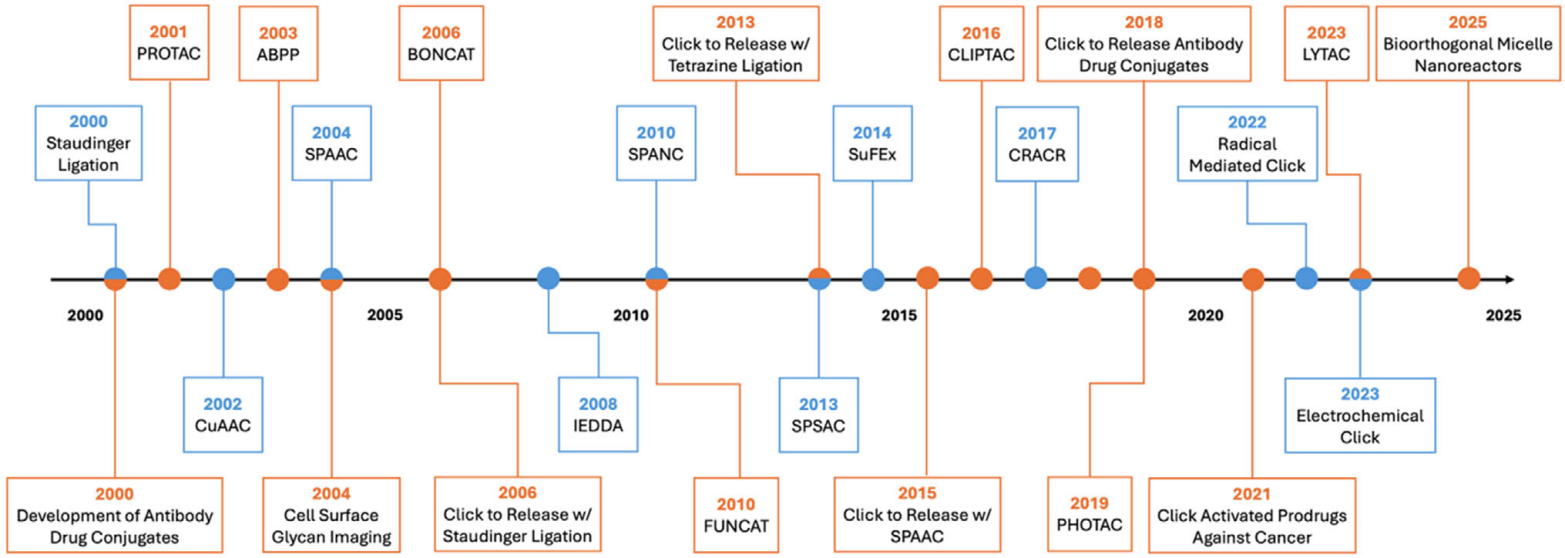
Timeline of advancements in bioorthogonal and click chemistry methodologies (blue) and their applications in biomedical and clinical research (orange).

The introduction of click chemistry and bioorthogonal chemistry changed the way that chemical synthesis was used to study biological systems and living organisms. As a result of their often‐overlapping concepts, reactions, and applications, click‐type and bioorthogonal chemistry are often mistaken for one another despite their clear differences; however, it is impossible to address one without recognizing the significance of the other. Notably, the plug‐and‐play nature of these two types of reactions in biological systems (especially living systems) makes them fit into one broader concept, biocompatible chemistry. The goal of this review is to summarize the most commonly used biocompatible reactions and their applications in reference to the fields of chemical biology research. Additionally, herein, we will discuss the development and expansion of the biocompatible chemistry toolbox over the past two and half decades (**Figure** **1**). Finally, we will summarize the advantages, limitations, and future outlooks of biocompatible chemistry for diagnostic applications.

### 1.1 Complexities of Chemical Reactions in Living Systems

Despite its intrinsic complexities, the nature of organic chemistry purposefully limits the potential outcomes of a given reaction scheme to simplify product isolation. Everything that is contained within the reaction flask is known and can be controlled to a degree; however, biology doesn’t always offer the same luxuries.^[^ ^6^ ^]^ The inherent properties of chemical reactions that occur within biological systems often require significantly more complex systems of action resulting in a significantly larger fundamental toolbox of what nature can synthesize. Additionally, evolution ensured that the chemical reactions that occur within living organisms exclusively promote its essential functions for survival, including energy production, metabolism, DNA replication, and protein synthesis resulting from highly specific, often enzyme‐catalyzed reactions.^[^ ^7^ ^]^

The integration of chemical reactions into biological systems is the result of billions of years of evolution making them extraordinarily complex and efficient. The precise chemical transformations required to maintain homeostasis within an organism have made them extremely sensitive to varying factors and exhibiting necessary properties such as reversibility, catalytic necessity, energy coupling, and even redundancy.^[^ ^8^ ^]^ Evolution has utilized the ideology of redundancy and degeneracy to add fail‐safes into living organisms resulting in highly efficient reactions that exhibit chemoselectivity unmatched to anything that could be produced in a lab.^[^ ^8^ ^,^ ^9^ ^]^ The complexity of the chemical framework that maintains biological function can be well summarized by chemical metabolism, signal transduction, and genetic code transcription and translation.

**Figure 2:**
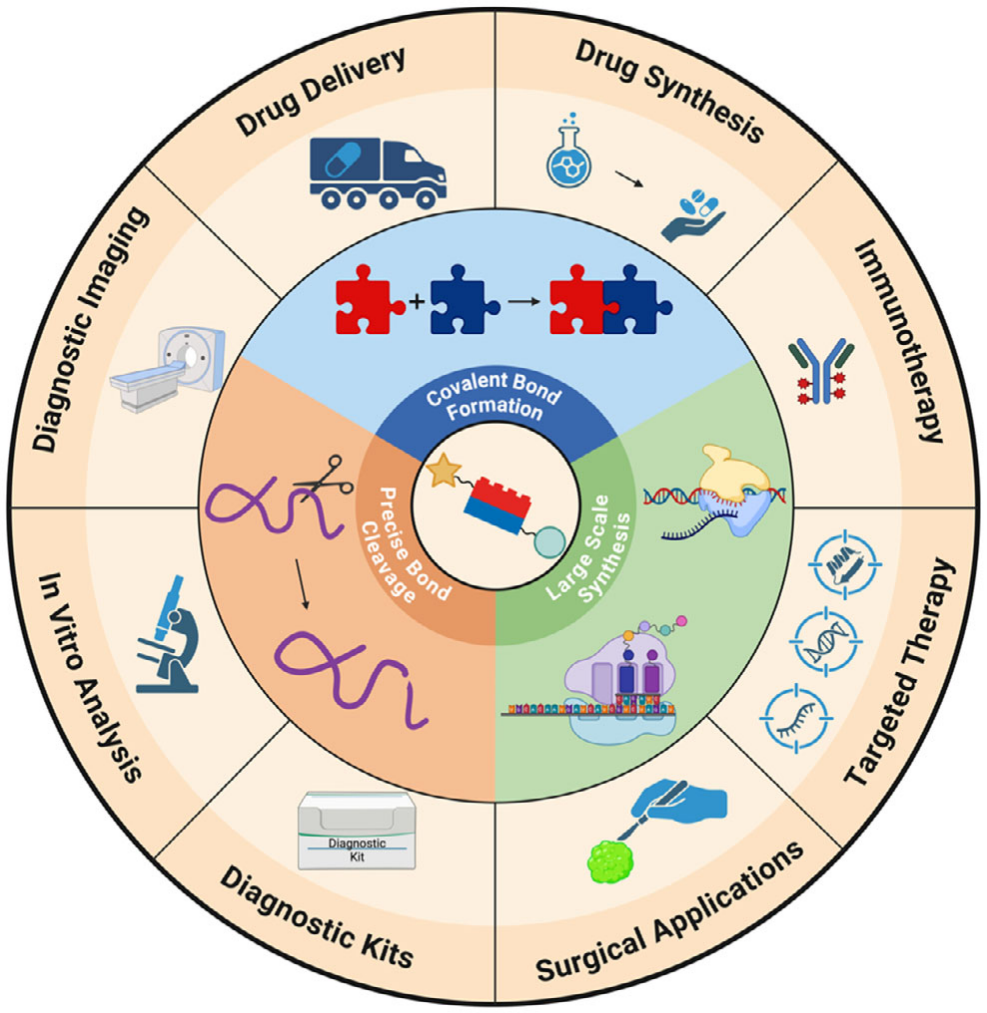
Fundamental principles and complexity of chemical reactions in living biological systems and the broad biomedical and clinical applications of modern biocompatible chemistry.

Endogenous chemical reactions in living organisms rely on selective control of covalent bond formation, precise bond cleavage, and the regulation of large‐scale macromolecule synthesis (**Figure** **2**). Metabolism consists of a system of interconnected cellular pathways ultimately used to break down macromolecules into metabolites to produce energy in the form of adenosine triphosphate, eliminate waste, perform molecular synthesis, and regulate homeostasis. For example, eukaryotes break down glucose and fatty acids introduced by food through a complex series of chemical reactions encompassed by glycolysis, acting as the primary source of cellular energy.^[^ ^10^ ^]^

Signal transduction is the process in which extracellular signals are transmitted into a cell to initiate an intracellular response. Signaling networks are mediated by ligand binding, the addition and removal of post translational modifications, and translocation of transcription mediating proteins to the nucleus. These processes are heavily regulated by a variety of highly specific chemical reactions to ensure proper cellular signaling. One example would be the addition and removal of phosphate groups on proteins by kinases and phosphatases respectively. Small mutations in the proteins that mediate these chemical reactions result in unregulated signaling pathways that have major implications in disease development.^[^ ^11^ ^]^

Large scale synthesis in living systems can be well defined by the intricate nuclear machinery encompassed by the central dogma. Both replication and transcription utilize polymerases to synthesize large nucleic acid polymers with high specificity through a dehydration reaction. Furthermore, the mRNA genetic code sequence is translated into polypeptides at the ribosome utilizing highly specific tRNA and synthetases. Additionally, synthesis of amide bonds in polypeptides are performed without need for protecting groups.^[^ ^11^ ^]^ Peptide synthesis can be performed by means of synthetic organic chemistry; however, this process involves the intricate protection and deprotection of reactive groups on amino acids, reducing the overall yield and requiring a tedious purification process.^[^ ^12^ ^]^

Metabolism, signal transduction, and polymerization have remained, for the most part, evolutionarily unchanged in living organisms and are considered essential parts of sustaining life. These are not the only chemical reactions that occur within living organisms; however, these accurately describe the selective, high yielding, and catalytically efficient reactions that make living systems so complex. The ability of biology to form and break covalent bonds and perform large scale polymerization reactions are feats that remain unmatched in the field of synthetic organic chemistry.^[^ ^13^ ^]^ Despite the discrepancy of what can be achieved by biology versus organic synthesis, chemists continue to mimic nature's complexity. This review aims to characterize a philosophy of synthetic chemistry that aims to mimic the efficiency of nature's chemical reactions with the primary goal of utilizing these reactions in living system. This philosophy has come to be known as biocompatible chemistry, which enables a wide range of applications in chemical biology and clinical studies (Figure 2).

### 1.2 Distinctive Characteristics of Click Chemistry and Bioorthogonal Chemistry

Despite the extremely complex nature of chemical reactions in biology, pioneer chemists, such as Sharpless, Bertozzi, and Meldal, have aimed to mimic the efficiency and selectivity of biological reactions. Such aspirations to follow nature have resulted in new chemistry‐based philosophies including click chemistry and bioorthogonal chemistry. Sharpless defined the field of ‘click chemistry’ as a set of high yielding, thermodynamically driven reactions that are highly stereospecific, but not necessarily enantiospecific. Subsequently, the reaction conditions should utilize simple, readily available functional groups in the starting materials while requiring benign solvents resulting in simple and nonchromatographic means of product isolation.^[^ ^2^ ^]^ The philosophy of click chemistry follows the idea of synthesis using simple molecular building blocks that rely on ‘spring loaded’ thermodynamically driven reactions, usually >20 kcal mol^−1^. This concept entails that these reactions are only thermodynamically driven under precise, sometimes catalytic, reaction conditions resulting in fast reaction kinetics and highly selective and highly stereospecific products.^[^ ^2^ ^,^ ^14^ ^]^

Bioorthogonal chemistry is a set of chemical reactions performed in complex biological environments without interfering with biomolecular or biochemical processes.^[^ ^1^ ^–^ ^5^ ^,^ ^15^ ^]^ Carolyn Bertozzi defined this concept based on the mathematical principle of ‘orthogonality’ describing multiple components that function independently without interfering with one another.^[^ ^16^ ^]^ For a reaction to be considered bioorthogonal, it must adhere to each of five fundamental principles in one way or another: 1) the reaction must occur in physiological environments; 2) the reaction must be high yielding and selective; 3) the reaction must not be affected by biological environments including water or endogenous cellular components; 4) the reaction must exhibit fast 2nd order kinetics; and 5) the reaction should involve unnatural functional groups. The concept of bioorthogonal chemistry uses synthetic reactions, typically performed in a lab, to study living organisms and cells. Whereas typical organic synthesis, click chemistry included, intends to create large amounts of material, bioorthogonal chemistry aims to study biomolecules in their native environments.^[^ ^15^ ^,^ ^17^ ^,^ ^18^ ^]^

The concepts of click chemistry and bioorthogonal chemistry are often used interchangeably due to the similarities in the concepts that define the reactions. Bioorthogonal chemistry, however, requires biocompatibility ensuring that chemical reactions have the potential to be used within living systems. It should be noted that many, but not all, click reactions are considered bioorthogonal under specific reaction conditions. Additionally, many chemical reactions are currently only considered click reactions as they lack one or more of the fundamental requirements that would give them bioorthogonal designation. Regardless, researchers are actively making headway to utilize click reactions in biocompatible applications. The quantitative threshold for what defines bioorthogonal chemical reactions remains relatively ambiguous; however, the above defined fundamental requirements hold true in that a reaction must, in part, meet all requirements to be considered bioorthogonal. This review intends to describe the contents of the current bioorthogonal and click reaction‐based biocompatible toolbox with the added goal of highlighting its plug‐and‐play nature and broad applications. Naturally, this review will not be able to encompass every click and bioorthogonal reaction presented in the literature, but it aims to cover the most widely known and commonly used reactions.

### 1.3 Incorporation of Bioorthogonal/Click Handles into Biomacromolecules

Biocompatible chemistry allows for the study and manipulation of biological systems using non‐native functionality. Bioorthogonal/click handles must be incorporated into biomolecules of interest and its complimentary reactive group on the exogenous probe, drug, or labeling technique.^[^ ^15^ ^]^

#### 1.3.1 Proteins

**Figure 3:**
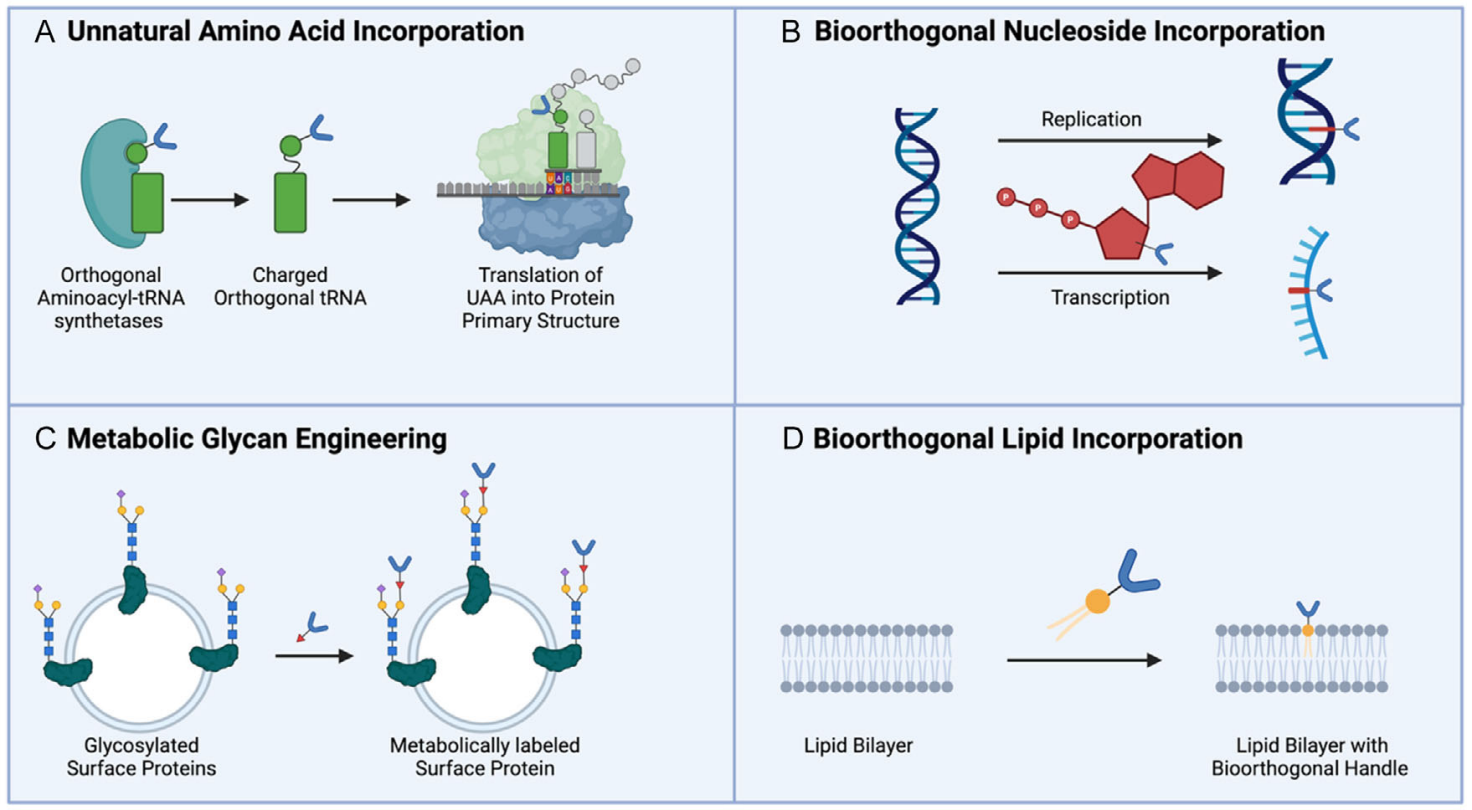
Incorporations of bioorthogonal handles into biomacromolecules. A) Unnatural amino acid incorporation into proteins. B) Incorporation of bioorthogonal nucleosides into nucleic acid polymers. C) Metabolic glycan labeling. D) Incorporation of bioorthogonal lipids.

The most common means of incorporating bioorthogonal handles into biomolecules is to integrate unnatural amino acids (UAA) into a protein primary structure (**Figure** **3**A).^[^ ^17^ ^,^ ^18^ ^]^ The incorporation of UAAs into a protein follow two general approaches: residue‐specific and site‐specific incorporation.^[^ ^19^ ^,^ ^20^ ^]^ Residue‐specific approaches substitute a natural amino acid with an unnatural one using a structural analog of the natural amino acid. This method has the benefit of using the native tRNA synthetase, hence, the UAA can simply be added to the culture allowing for its incorporation into proteins. A hindrance of this method, however, is that the UAA can replace the natural amino acid at several sites on a protein, some of which being buried in the protein's tertiary structure, and hence, inaccessible to bioorthogonal modification. Additionally, the UAA could be incorporated into various proteins making it difficult to isolate and study a single protein of interest or class of proteins.^[^ ^20^ ^]^

Site‐specific UAA incorporating also takes advantage of the cell's natural translational machinery; however, it relies on the manipulation of nonsense or stop codon. Stop codons, including the amber, ochre, and opal codons, have the natural function of terminating the translation process through recognition from release factors. This machinery can be manipulated by using an artificially evolved orthogonal tRNA synthetase that competes with release factors to add a given UAA rather than terminating translation at the place of the stop codon. This gives the benefit of incorporating a UAA at a specific site on a protein of interest. This method also has its drawbacks as it requires the additional step of creating an orthogonal tRNA synthetase resulting in a longer research and development process for UAA incorporation.^[^ ^21^, ^22^ ^–^ ^23^ ^]^

#### 1.3.2 Nucleic Acids

The addition of click handle into nucleic acid sequences (DNA and RNA) is relatively straightforward in reference to protein incorporation. A common method of incorporating bioorthogonality into nucleic acids is to metabolically incorporate modified nucleoside triphosphate groups. For purine‐based nucleosides, modification often occurs at the 2 or 6 positions on the ring and for pyrimidine‐based nucleosides at the 5 positions. Azide‐modifications have also been incorporated into both ribose and deoxyribose of nucleoside triphosphates at the 2′, 3′, 4′, and 5′ positions on the sugar rings.^[^ ^24^ ^]^ Additionally, alkenyl deoxyribonucleosides and ribonucleosides have also been used for the incorporation of bioorthogonal handles into DNA and RNA for Diels–Alder type bioorthogonal modification.^[^ ^24^, ^25^ ^–^ ^26^ ^]^ Modified nucleoside triphosphates are easily incorporated into DNA and RNA through a cell's normal replication and transcription machinery (Figure 3B). A downside of this method is that modified nucleosides can replace canonical nucleosides at multiple sites within nucleic acid polymers. As a result, the current literature has shown limited clinical uses for nucleic acid bioorthogonality with its primary uses being labeling and imaging.^[^ ^15^ ^,^ ^24^, ^25^, ^26^ ^–^ ^27^ ^]^

#### 1.3.3 Glycans

Glycosylation patterns can serve as an indicator of a variety of disease states. Studying glycans has proven to be an extremely difficult feat as they are not genetically encoded, meaning that the same chemical biology techniques used for studying nucleic acids and proteins are not applicable. Bioorthogonal chemistry has served as a means of bypassing this limitation.^[^ ^15^ ^,^ ^28^ ^]^ Bertozzi et al.^[^ ^29^ ^]^ developed a method of incorporating bioorthogonal handles into glycan structures called metabolic oligosaccharide engineering (MOE). By treating a cell with unnatural monosaccharide subunits containing bioorthogonal functionality, the cell's glycan biosynthetic machinery can then subsequently incorporate it into the nonspecific glycoconjugates to act as a reporter tag (Figure 3C).^[^ ^28^, ^29^ ^–^ ^30^ ^]^ MOE follows a similar methodology to residue‐specific UAA integration in proteins as it utilizes unnatural precursors containing a reactive group that is inert to biological processes, allowing for later bioorthogonal modification. Naturally, it also has a lot of the same limitations as it is not orthogonally incorporated resulting in nonspecific integration throughout a cell.

#### 1.3.4 Lipids

Lipids exhibit similar difficulties to glycans for studying within the biological system. Additionally, bioorthogonal labeling of lipids have proposed many challenges due to the vast number of functions they perform in the cell. The most common means of bioorthogonal handle incorporation into lipids is the use of fatty acid analogs (Figure 3D). These analogs often feature an alkyne or azide added to the terminal methyl group of lipid species including fatty acids, sterols, phospholipids, and sphingolipids.^[^ ^28^ ^,^ ^31^ ^,^ ^32^ ^]^ The bioorthogonal handles that can be added to lipids are limited to relatively small functional groups as it is often imperative that the function of the nonpolar tail remains unchanged. Additionally, the variety of lipids to which fatty acid analogs could be incorporated makes studying and targeting specific lipid interaction difficult. Researchers have addressed these issues by attaching a bioorthogonal handle to the tail or head group and a photoreactive cross‐linking group on the tail of the analog showing potential versatility to visualize, crosslink, and even exploit selective integration into lipids.^[^ ^33^ ^]^ Despite recent advancements, lipids remain one of the least studied biomacromolecules using bioorthogonal methods.

## 2 Main Types of Biocompatible Reactions

**Figure 4:**
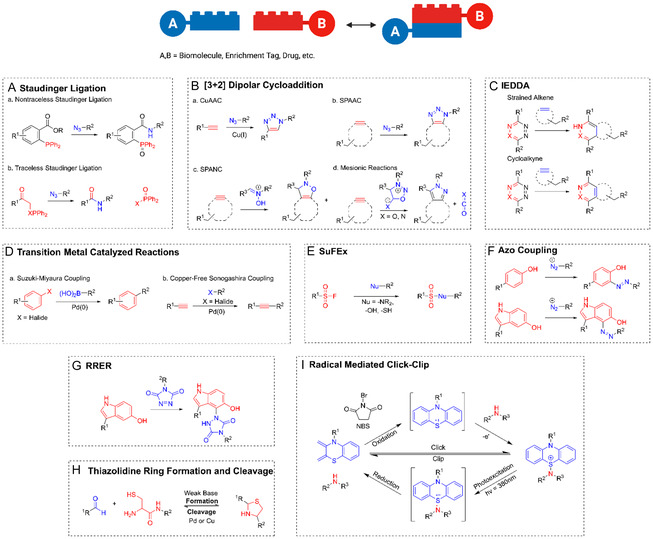
A–I) Summary of the representative biocompatible chemistry discussed in this review, including Staudinger ligation, CuAAC, IEDDA, SuFEx, RRER, and so on, which contributes to forming a plug‐and‐play toolbox for chemical biology research.

The current scope of bioorthogonal and click reactions encompassed in the biocompatible toolbox are described below (**Figure** **4**). This set of chemical reactions is summarized in **Table** **1** in order of when they are discussed in the review.

**Table 1:** Summary table comparing the representative bioorthogonal and click chemistry discussed throughout the review, outlining key differences including catalysts, substrates, reaction rates, and disadvantages.

Reaction	Catalyst/additive	Substrates	Second‐order rate constant [M^−1^ s^−1^]	Disadvantages
Staudinger ligation	None	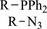	10^−3^	Slow reaction kinetics
CuAAC	Copper	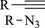	10^0^–10^2^	Toxicity of Cu
SPAAC	None	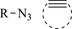	10^−2^–10^0^	Bulky hydrophobic group, reactivity with thiols
SPANC	None	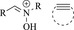	10^0^–10^1^	Bulky hydrophobic group,Instability of nitrones
SPSAC	None	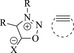	10^0^–10^1^	Bulky hydrophobic group,Instability of sydnones
IEDDA	None	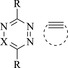	Up to 10^6^	Bulky hydrophobic group
Suzuki–Miyaura coupling	Palladium	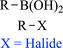	10^−1^–10^0^	Toxicity of Pd, Boronic acid oxidation
Copper‐free Sonogashira coupling	Palladium	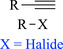	10^−1^–10^0^	Toxicity of Pd
SuFEx	None	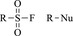	10^−1^–10^0^	Lacks selectivity
Photoclick	UV Light	Varying	10^1^	Requires UV Light
CRACR	None	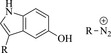	10^1^–10^4^	Tyrosine reactivity
RRER	None	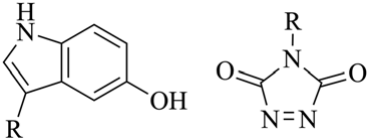	10^1^–10^4^	Tyrosine reactivity

### 2.1 Staudinger Ligation

#### 2.1.1 Nontraceless Staudinger Ligation

The Staudinger reaction was first discovered by Hermann Staudinger in 1919 using nucleophilic phosphanes and azides to yield carboxamide and phosphine oxide products (Figure 4A.a).^[^ ^26^ ^]^ In 2000, Bertozzi et al.^[^ ^5^ ^]^ modified this reaction to work bioorthogonally becoming known as the nontraceless Staudinger ligation. An endogenous azide is used with an aryl phosphane which takes advantage of an electrophilic trap (methyl ester) to induce amide formation at the methyl ester. The ‘nontraceless’ denomination results from the aryl ring that connects the endogenous and exogenous groups and the phosphine oxide remaining unreleased from the product. The first example of bioorthogonality used the nontraceless Staudinger ligation to label synthetic azidosugars on the cell surface. Bertozzi predicted, based on the results, that this technique could be expanded to probe the interior of the cell.^[^ ^5^ ^,^ ^26^ ^]^

#### 2.1.2 Traceless Staudinger Ligation

Shortly after the introduction of the nontraceless Staudinger ligation, Bertozzi^[^ ^34^ ^]^ and Raines^[^ ^35^ ^]^ introduced the traceless Staudinger Ligation. The traceless Staudinger ligation takes advantage of a cleavable heteroatom linker that connects the phosphane to the electrophilic trap resulting in an amide product that does not contain the phosphine oxide scar (Figure 4A.b.). Traceless Staudinger ligation has been extensively used for peptide/protein conjugation as it naturally incorporates an amide into the backbone. The N‐terminal amino acid of one peptide is azide‐functionalized while another peptide has a c‐terminal phosphinothioester allowing for the seamless conjugation of the two.^[^ ^15^ ^,^ ^26^ ^]^

The Staudinger Ligation was the first bioorthogonal reaction showing relatively versatile capabilities in chemical biology; specifically in labeling, ligation, and bioconjugation. It utilizes a small azide functional group which can be easily incorporated into a variety of biomacromolecules. Additionally, the unique reactivity profile of nucleophilic phosphines with azides results in good selectivity. The Staudinger ligation, however, displays relatively slow 2nd order reaction kinetics (10^−3^ M^−1^s^−1^) leading to inefficient labeling and ligation.^[^ ^13^ ^,^ ^36^ ^]^ Additionally, phosphanes are prone to oxidation, hence, high concentrations of phosphanes are required, however, borane‐protected phosphanes have shown promise in addressing this issue.^[^ ^37^ ^]^ The iminophosphorane intermediate can also be prematurely hydrolyzed resulting in reduced phosphorylamines or amines making the reaction pH dependent in aqueous media.^[^ ^15^ ^,^ ^26^ ^,^ ^37^, ^38^ ^–^ ^39^ ^]^ Though still a useful bioorthogonal reaction in many ways, the Staudinger ligation has become relatively incomparable to bioorthogonal reactions that proceeded it.

### 2.2 [3 + 2] Cycloaddition

#### 2.2.1 CuAAC

A trademark reaction within the bioorthogonal toolbox is CuAAC. Discovered in 2001 independently by Sharpless^[^ ^3^ ^]^ and Meldal,^[^ ^4^ ^]^ CuAAC has become the most broadly used bioorthogonal reaction in chemical biology, drug discovery, and drug delivery. CuAAC utilizes an organoazide and a terminal alkyne to produce a 1,2,3‐triazole (Figure 4B.a). CuAAC is an advancement of the Huisgen 1,3‐dipolar cycloaddition, discovered by Rolf Huisgen in the 1960s, used in organic synthesis to create 5‐membered heterocycles. The uncatalyzed Huisgen reaction requires elevated temperatures and long reaction times in addition to poor regioselectivity between the 1,4‐ and 1,5‐products.^[^ ^40^ ^,^ ^41^ ^]^ The introduction of a copper (I) catalyst broadened the scope of applications for this reaction as it solely affords the 1,4‐regioisomer and significantly accelerates the reaction rate. Additionally, CuAAC can be performed at ambient temperatures to afford high yields.^[^ ^40^ ^]^

CuAAC exhibits very fast 2nd order rate constants (10^0^–10^3^ M^−1^s^−1^), regioselectivity, and high yields meeting all the criteria of a click reaction. Additionally, the reaction is insensitive to parameters such as aqueous solvents, pH (4–12), and avoids functional group interference qualifying it as an ideal candidate to be used bioorthogonally.^[^ ^13^ ^,^ ^40^ ^]^ Sharpless and Finn^[^ ^42^ ^]^ were the first to use CuAAC bioorthogonally to bioconjugate the exterior of the Cowpea mosaic virus. Reactive lysine or cysteine residues were decorated with azides or alkynes that were ligated with fluorescein derivatives containing the complementary reactive group allowing for visualization. Another notable finding from this study was that the addition of tris(triazolyl) amine, specifically tris(benzyltriazolylmethyl)amine (TBTA), as a ligand greatly increased the reaction rate while also deterring Cu(I) oxidation in aqueous media.^[^ ^42^ ^]^

An early issue with the translation of CuAAC into a bioorthogonal applications was that copper ions exhibit cytotoxic effects in cells as they can chelated to amino acid residues changing their folding pattens, structure, and activity. Additionally, Cu(I) can be oxidized into Cu(II) which catalyzes the formation of reactive oxygen species (ROS) in cellular environments.^[^ ^43^ ^,^ ^44^ ^]^ To allow for suitable in vivo studies using CuAAC, extensive research efforts were placed into the development of ligands that would stabilize the copper catalyst in cellular environments. These efforts afforded a variety of promising ligands with the most promising being tris amine ligands that came as derivatives of TBTA.^[^ ^42^ ^,^ ^44^, ^45^, ^46^ ^–^ ^47^ ^]^ It should be noted that other effective ligands have been developed for use in CuAAC, however, the tris amine derivatives exhibit the unique ability to both reduce the toxicity of Cu catalysts and enhance the reaction rate of CuAAC reactions with the best example being TBTA.^[^ ^47^ ^]^

#### 2.2.2 Strain‐Promoted Azide–Alkyne Cycloaddition (SPAAC)

One means of addressing the initial toxicity issues related to CuAAC was to develop copper‐free reactions. Strained cycloalkynes emerged as a promising alternative resulting from their similar reactivity with azides to afford 1,2,3‐triazole rings (Figure 4B.b). Bertozzi et al.^[^ ^48^ ^]^ were the first show that SPAAC could be used bioorthogonally. By metabolically labeling the cell surface with azide‐modified glycans, Bertozzi showed that SPAAC could selectively modify biomolecules in vitro and on living cells with no apparent toxicity.^[^ ^48^ ^]^

Early cyclooctynes struggled with relatively slow reaction kinetics resulting in reagent being required in large excess, long incubation times, and overall showed poor viability. Research efforts were hence placed into the discovery of more reactive cycloalkynes that could display reactivity comparable to CuAAC. Two major classes of cyclooctynes emerges with promising reactivity profiles: aliphatic and dibenzoannulated cyclooctynes. Bertozzi et al. provided many of the early examples of SPAAC, with most being aliphatic cyclooctynes. Bertozzi et al.^[^ ^48^ ^,^ ^49^ ^]^ reported the first generation cyclooctyne (OCT) as an azide reactive partner with relatively low second order reaction kinetics (0.0012 M^−1 ^s^−1^) featuring an electron donating ether group on C3.

Significant developments in cyclooctynes analogs leveraged ring strain, lipophilicity, and synthetic availability. Dibenzocyclooctyne (DIBO, 0.17 M^−1^s^−1^),^[^ ^50^ ^]^ dibenzoazacyclooctyne (DIBAC, 0.31 M^−1 ^s^−1^),^[^ ^51^ ^]^ and bicyclononyne (BCN, 0.14 M^−1^s^−1^)^[^ ^52^ ^,^ ^53^ ^]^ emerged as the most commonly used SPAAC cyclooctyne reagents for their sufficient reactivity and synthetic accessibility.^[^ ^49^ ^]^ Advantages of SPAAC include the use of small functionality (azides) that can be easily incorporated into biomacromolecules. It also does not rely on a catalyst as reaction kinetics are driven by the inherent ring strain of the cyclooctyne reaction partner. Additionally, when used bioorthogonally, SPAAC second‐order reaction kinetics are reported to be moderately high (10^−1^–10^1 ^M^−1 ^s^−1^). Limitations for SPAAC include the use of a large reactive partner in cyclooctynes with the addition of nonspecific alkyne reactivity with nucleophilic cysteine. Additionally, SPAAC struggles with regiospecificity creating a mixture of 1,4‐products which, for its primary uses, is generally a nonfactor.^[^ ^13^ ^,^ ^15^ ^]^ Overall, the development of a strain‐promoted [3 + 2] cycloaddition resulted in the development of a variety of other bioorthogonal methods that rely on the same principles.

#### 2.2.3 Strain‐Promoted Alkyne–Nitrone Cycloaddition (SPANC)

Building off the work of SPAAC, nitrones served a means of multifunctional bioorthogonality sparking the development of SPANC. Nitrones possess three potential modification sites allowing for increase versatility in comparison to SPAAC to allow for stereoelectronic control and manipulation of ring strain (within cyclic nitrones) to drive reaction rates. Cyclooctynes react with nitrones in a [3 + 2] cycloaddition to yield isoxazole products (Figure 4B.c). As nitrones are another class of 1,3‐dipoles, are nonnative in biological environments, offer simple synthetic means, and exhibit high selectivity with the addition of structural versatility, they became a promising avenue for bioorthogonal development.^[^ ^54^ ^]^

The van Delft^[^ ^55^ ^]^ group was the first to report SPANC, utilizing acyclic nitrones with DIBO and DIBAC resulting in increased second‐order rate constants of 10^−1^ and 10^0 ^M^−1 ^s^−1^ respectively. The method showed success in modification of both synthetic peptides and a short native protein (chemokine interleukin‐8, 72 amino acids).^[^ ^55^ ^]^ The results of this work were extremely promising for bioorthogonal viability of SPANC. Early reports from Pezacki et al.^[^ ^56^ ^]^ showed the reactivity of DIBO with both acylic and endocyclic nitrones displaying significantly increased second order reaction kinetics (10^−1 ^M^−1 ^s^−1^), 25 times faster than its benzyl azide counterpart. Subsequent reports with BCN^[^ ^57^ ^]^ followed the same trend with a second‐order rate constant of 10^−1 ^M^−1 ^s^−1^ respectively.

SPANC was developed as a means of building off the initial works of SPAAC utilizing variable functionality to increase reaction rate and solubility in strain‐promoted reactions. It offers the advantage of significantly increased reaction rates (10^0^–10^1 ^M^−1 ^s^−1^) in bioorthogonal applications as a result of the highly tunable multifunctional reactivity offered by nitrone substrates. Additionally, it does not rely on a catalysts and reaction kinetics are driven by the inherent ring strain of the cyclooctyne and nitrone substrates. Limitations for SPANC, like SPAAC, include the use of large reactive partners in cyclooctynes. Furthermore, nitrones can suffer from instability issues that limit their in vivo and in vitro applications. Finally, SPANC also struggles with regioselectivity, creating a mixture of products.^[^ ^13^ ^,^ ^15^ ^]^

#### 2.2.4 Strain‐Promoted Sydnone–Alkyne Cycloaddition (SPSAC)

Since the introduction of strain‐promoted cycloaddition, many adaptations have been reported. Most of these have focused on aspects that aim to improve upon kinetics, stability, versatility, and overall expand the toolbox of strain‐promoted reactivity (Figure 4B.d). Similar to SPANC, many strain‐promoted reactions have focused on investigating different reactivity partners with strained cyclooctynes. Other examples of 1,3‐dipoles can be classified as mesoionic compounds, specifically sydnones.^[^ ^17^ ^]^ Taran et al.^[^ ^58^ ^]^ were the first to report reactivity of sydnones with nonstrained alkynes in the presence of a copper catalyst. Chin et al.^[^ ^59^ ^]^ would build upon this work to develop SPSAC. This reaction occurs through a [3 + 2] cycloaddition followed by a retro‐Diels–Alder type mechanism to form the pyrazole product with CO_2_ as a byproduct. SPSAC showed comparable reaction kinetics with BCN to SPAAC displaying a second‐order rate constant of 0.054 M^−1 ^s^−1^.^[^ ^59^ ^,^ ^60^ ^]^ Furthermore, the use of halogeno‐sydnones significantly increased second‐order rate constants to 42 M^−1^s^−1^ for BCN.^[^ ^60^ ^]^ While SPSAC offers a promising means of advancement on SPAAC and SPANC, its scope remains extremely limited and its substrates struggle with aqueous solubility.

### 2.3 [4 + 2] Cycloaddition

Inverse electron‐demand Diels–Alder (IEDDA) reactions are a class of click and bioorthogonal reactions that undergo a [4 + 2] cycloaddition (Figure 4C). The reaction utilizes an electron‐deficient diene and an electron‐rich dienophile undergoing a concerted bicyclooctane‐type transition state to form a six‐membered ring. Contrary to traditional Diels–Alder reaction, IEDDA reactions involves a reversal of electronic properties, with an electron‐poor diene (e.g., tetrazines, triazines) and an electron‐rich dienophile (e.g., trans‐cyclooctene, norbornene). Naturally, this flips the electronic effects of traditional Diels–Alder reaction with IEDDA electronics being characterized by the addition of an electron withdrawing group to the diene lowering the lowest unoccupied molecular orbital (LUMO) and an electron donating group to the dienophile raising the highest occupied molecular orbital (HOMO) allowing for favorable reactivity. This reaction is characterized by extremely fast reaction kinetics making it one of the fastest bioorthogonal reactions available. Additionally, IEDDA reactions are highly selective and biocompatible, enabling bioorthogonal applications in complex biological environments without interfering with native processes. The unique combination of speed, selectivity, and versatility has established IEDDA reactions as a cornerstone of bioorthogonal chemistry.^[^ ^17^ ^,^ ^61^ ^]^

The first example of IEDDA was presented by Sauer^[^ ^62^ ^]^ in 1990 who investigated the exceptionally fast reaction kinetics between an electron‐deficient tetrazine diene and various strained and nonstrained alkene dienophile derivatives. Two di‐substituted tetrazine derivatives were used in the substrate scope; one containing a moderately electron‐withdrawing methoxycarbonyl groups and the other containing strong electron‐withdrawing trifluoromethyl groups. Naturally, stronger electron‐withdrawing capabilities from the electron‐deficient diene generally increased reactivity of the diene substrate. IEDDA reactions were performed in organic media producing second‐order rate constants ranging from 10^4^ to 10^14 ^M^−1 ^s^−1^ with trans‐cyclooctene (TCO) displaying the highest reaction kinetics.^[^ ^62^ ^]^

Fox et al.^[^ ^63^ ^]^ reported the first bioorthogonal use of IEDDA in 2008, giving it the name tetrazine ligation. They reported a second‐order rate constant of 2000 M^−1 ^s^−1^ in 9:1 methanol:water and showed success in protein labeling giving promise to its use in vivo applications.^[^ ^63^ ^]^ Much early research focused on optimizing the diene to delicately balance reactivity and stability in aqueous media resulting in a variety of dipyridyl tetrazine derivatives.^[^ ^64^, ^65^ ^–^ ^66^ ^]^ Additionally, it was found that triazines displayed increased stability in reference to tetrazines, however, this improvement in stability had a contrasting effect on reactivity.^[^ ^67^, ^68^ ^–^ ^69^ ^]^ Reactivity of cycloalkyne dienophiles have been explored as an expansion of strain‐promoted cycloaddition reactions. Wang et al. investigated the reactivity of OCT and BCN with tetrazine diene derivatives.^[^ ^70^ ^]^ They reported second‐order rate constants of OCT and BCN with dipyridyl tetrazine (pyTz) in MeOH to be 2.0 and 44.8 M^−1 ^s^−1^, respectively.^[^ ^70^ ^]^ Overall, uses of cyclooctynes for IEDDA type reaction are limited in comparison to TCO, however, they can be more stable alternatives to TCO derivatives if reaction rate is not an issue. Additionially, nonstrained alkenes have also been shown to be viable for IEDDA reactivity as an effective means of pretargeting in live cell imaging.^[^ ^61^ ^,^ ^71^, ^72^, ^73^, ^74^ ^–^ ^75^ ^]^ Additionally, Park et al.^[^ ^76^ ^]^ used tetrazine ligation at a proteome‐wide scale to label Bruton's tyrosine kinase proteins in live cells. Results showed that tetrazine derivatives exhibit structure‐dependent proteome reactivity that can significantly influence accuracy of fluorescent protein imaging.^[^ ^76^ ^]^

Tetrazine ligation offers several advantages including its extremely fast second‐order kinetics, with rate constants reaching up to 10^6^ M^−1^s^−1^. Additionally, the reaction proceeds without the need for a catalyst. These advantages have made it nearly ideal for live cell studies, however, the high reactivity of tetrazines result in varying stability in aqueous media. In addition, their functionality may be compromised in the presence of thiols resulting in minimal nonselective reactivity. These factors can restrict their use for studying biomolecules in their native environments, regardless, IEDDA has emerged as one of the best methods of cellular investigations in chemical biology.^[^ ^13^ ^,^ ^15^ ^,^ ^61^ ^]^

### 2.4 Transition Metal‐Catalyzed Bioconjugation

The significance of carbon–carbon bond formation has served as the driving force for many aspects of research in organic synthesis for many decades. Cross‐coupling reactions became staple organic reactions with the Suzuki–Miyaura (Figure 4D.a)^[^ ^77^ ^]^ and copper‐free Sonogashira cross couplings (Figure 4D.b)^[^ ^78^ ^]^ emerging as promising palladium‐catalyzed methods of C─C bond formation. With both reactions exhibiting high selectivity in physiological conditions and good functional group tolerances, they have emerged as a promising class of reactions to be translated for bioorthogonal applications.^[^ ^13^ ^,^ ^79^ ^,^ ^80^ ^]^

The first example of palladium being used bioorthogonally was in 2010 by Ahn et al.^[^ ^81^ ^]^ who used PdCl_2_ to catalyze depropargylation of an inactive fluorophore to activate it in vivo zebrafish studies. Though this initial experiment did not utilize Pd for purposes of bioconjugation, it opened the door for the use of transition metals to perform selective chemical transformations in living systems. Bradley et al.^[^ ^82^ ^]^ reported the use of Pd^0^ nanoparticles to catalyze bioorthogonal reactions, such as allylcarbamate cleavage and Suzuki–Miyaura cross‐coupling reactions to create active fluorophores in cells. Similar methods were used to perform Sonogashira coupling enabling fuctionization of homopropargylglycine (HPG)‐encoded ubiquitin protein in aqueous medium and in *E. coli* cells further demonstrating the potential use of Pd‐catalyzed reaction in bioorthogonal application.^[^ ^83^ ^]^

Although previously considered a prohibitively expensive and chemically inert metal, gold has recently emerged demonstrating high catalytic activity in various organic transformations and recently displaying promise in bioorthogonal applications.^[^ ^84^ ^]^ Tsubokura et al.^[^ ^85^ ^]^ demonstrated the first transition metal‐catalyzed covalent bond formation in mice studies. Using albumin as both a carrier and targeting vehicle for metal complexes. Albumin surfaces were modified with N‐glycans allowing for organ‐selective accumulation. Propargylic esters, activated by a Au(III) catalyst, act as acyl donors for glycan‐targeted organs. Activation enabled target‐selective labeling via nucleophilic exchange with amines present on surface proteins of the targeted tissue.^[^ ^85^ ^]^

Transition metals have also been used as a means of selective bond cleavage of propargyl, allylic, and vinylic heteroatoms and carbamates. The first report of a transition‐metal‐catalyzed bioorthogonal cleavage reaction was by Megger et al.^[^ ^86^ ^]^ reporting Ru‐mediated reprotection of allyl carbamate to activate a fluorescent dye in live cells. Koide et al.^[^ ^87^ ^]^ would report a similar experiment in 2007 where they used Pd to trigger the deallylation of fluorescent probes to which Ahn et al.^[^ ^88^ ^]^ would later transition into a zebrafish in vivo model. Chen et al.^[^ ^89^ ^]^ would build on this work in 2014 using Pd to catalyze the deprotection of a catalytic Lys residue on a protein in living cells. While palladium remains the most studied transition metal used for biocompatible reactions, ruthenium, copper, gold, and platinum have all shown promising biocompatible applications.^[^ ^90^ ^]^

Despite the efficiency and highly selective nature of transition metal‐catalyzed reactions, they pose the limitation of cytotoxicity due to protein–metal complexes^[^ ^91^ ^,^ ^92^ ^]^ and the formation of ROS.^[^ ^13^ ^]^ Metallopeptide complexes have shown promise in addresses protein–metal complex formation, however, they struggle with solubility and cell permeability.^[^ ^93^ ^]^ Ligands have also been used to coordinate metals,^[^ ^94^ ^]^ however, many Pd‐specific ligands are instable in aqueous media.^[^ ^13^ ^]^

### 2.5 Sulfur (VI) Fluoride Exchange (SuFEx)

SuFEx chemistry was first reported in 2014 by Sharpless et al.^[^ ^95^ ^]^ who found that under unique conditions S─F bonds can convert from a strong covalent bond to a leaving group. Interactions of H^+^ and R_3_Si^+^ assist in close proximity under strict kinetic and spatial constraints to allow for this unique electrophilic reactivity. Sharpless further demonstrated that SuFEx chemistry could be applied with a diverse scope of nucleophiles including amines, alcohols, and thiols (Figure 4E). Additionally, this unique reactivity was further extended to amino acids containing corresponding nucleophiles including cysteine, lysine, histidine, tyrosine, serene, and threonine. The unique reactivity of S─F bonds is only achieved through a variety of discrete molecular plugins or SuFExable‐hubs. There are five discrete classes of SuFEx connective hubs including sulfonyl fluorides, sulfuryl fluoride, thionyl tetrafluoride, ethanesulfonyl fluoride, and 1‐bromoethane‐1‐sulfonyl fluoride. Due to its generally unreactive nature of S─F bonds, unless specific activation conditions are met, it has incredible potential for bioorthogonal adaptation.^[^ ^95^, ^96^ ^–^ ^97^ ^]^

SuFEx reactions exhibit many of the necessary qualities of a bioorthogonal reaction; working exceptionally well in aqueous media, exhibiting sufficiently fast second‐order reaction rate constants (10^−1^–10^0 ^M^−1 ^s^−1^), utilizing an unnatural functional group, and is very high yielding. However, it has not gained bioorthogonal status as it lacks high selectivity as SuFEx hubs can react with multiple nucleophilic residues within a cell. Regardless, SuFEx can still be used in chemical biology applications, such as activity‐based protein profiling (ABPP), with its lack of selectivity acting as a potential advantage in this application.^[^ ^95^ ^,^ ^97^ ^]^ Though still in its adolescence, SuFEx has the potential to be the next great bioorthogonal reaction with its great advantage being its ability to study biomacromolecules in their native environments without the need for handle incorporation.

### 2.6 Other Types of Biocompatible Chemistry

Photoclick chemistry has emerged as an adaptive methodology of many of the previously discussed reactions using light to overcome reaction barriers enabling rapid, selective, and efficient covalent bond formation under mild conditions, often with spatiotemporal control. The methodology requires photoactive reagents to which irradiation with UV or visible light will generate reactive intermediates which can further undergo click‐type reactions. One advantage of photoclick methodologies is the high tunability of reaction kinetics based on light intensity, wavelength, and the concentration of reactions. Most recently, Feng et al.^[^ ^98^ ^]^ reported a novel light‐promoted bioorthogonal reaction to which they coined the name light‐promoted bioorthogonal multifunctionalized molecular recombination (LBMR). LBMR was reported to facilitate the synthesis of polysubstituted pyrroles from isoxazole‐3‐carboxylate and isoxazole‐3‐carboxylic acid derivatives. This reaction displayed successful labeling and imaging potentials within cancer cell lines and zebrafish models.^[^ ^98^ ^]^ Second order reaction rate constants of photoclick methodologies typically range around 10^1 ^M^−1 ^s^−1^ dependent on earlier expressed factors. Despite being highly versatile, downsides of photoclick chemistry lie in its use of UV light limiting its uses for in vivo applications.^[^ ^13^ ^,^ ^99^ ^]^

Another click‐based bioconjugation is chemoselective rapid azo‐coupling reaction (CRACR) utilizing an adaptation of a well‐known methodology in a chemoselective manner to be used in biological applications. CRACR utilizes aryl diazonium salts and electron‐rich aromatic compounds enabling the formation of azo bonds effectively conjugating to activated indoles and tyrosine in biological environments (Figure 4F).^[^ ^100^ ^]^ Triazolinedione (TAD) has also been recently used for click‐type bioconjugations. TAD reagents were initially developed to protect indoles in total synthesis of natural products through a reversible ene‐type reaction of the diazo structure in TAD and the C2 atom of the indole (Figure 4G).^[^ ^101^ ^]^ Similar to CRACR in its reaction kinetics, selectivity, and biocompatibility, the TAD ene‐type reaction has also been adapted for bioorthogonal labeling of tyrosine, however, recent findings have revealed that indole scaffolds become the preferred substrate under acidic conditions.^[^ ^102^ ^,^ ^103^ ^]^ CRACR and ene‐type reactions offer fast kinetics (10^1^–10^4 ^M^−1 ^s^−1^) while operating under mild, aqueous conditions, however, its lack of specificity and reliance of pH limits its ability to be truly bioorthogonal by classical definitions.^[^ ^100^ ^,^ ^102^ ^,^ ^103^ ^]^ Gouin et al.^[^ ^103^, ^104^ ^–^ ^105^ ^]^ has recently reported an electrochemical, tyrosine specific biocompatible click‐type reaction (eY‐click) reaction involving the use of electroactive probes to manipulate the tunability of kinetics and selectivity in ene‐type reactions. By leveraging the principles of click chemistry and electrochemistry, rapid and efficient methods for labeling the surface proteins of viruses, bacteria, and cells have been successfully achieved.^[^ ^103^ ^–^ ^105^ ^]^

Furthermore, radical‐mediated click‐type chemistry was first proposed by Scanlan et al.^[^ ^106^ ^]^ in 2022 as a means of bioconjugation through a thiol‐ene type reaction. Recently, Zhang et al.^[^ ^107^ ^]^ reported a radical‐mediated click‐clip reaction of phenothiazines and amines through the formation (click) and cleavage (clip) of sulfimine bonds (Figure 4I). Sulfilimine bond formation can be facilitated by N‐bromosuccinimide (NBS) to induce phenothiazine oxidation and subsequent coupling to the amine substrate. Additionally, photexcitation at 380 nm can induce reduction of the sulfimine bond affording the initial phenothiazine and amine starting materials.^[^ ^107^ ^]^ This work highlights the direction in which modern click‐based chemistry is headed with the end goal of reversible bioconjugation.

Finally, in 1995 Zhang and Tam^[^ ^108^ ^]^ described a means of protein bioconjugation through thiazolidine formation where a 1,2‐aminothiol moiety from an N‐terminal cysteine reacts with an aldehyde to form a stable thiazolidine ring. This reaction provides an alternate method for linking synthetic peptides and proteins to other molecules under mild conditions in aqueous media. The thiazolidine moiety has since been used to facilitate bond cleavage in a bioorthogonal manner. Brik et al.^[^ ^109^ ^]^ were among the first to use this method using palladium complexes to cleave proteins at specific sites that have been modified with a thiazolidine backbone. Additionally, Bi et al.^[^ ^110^ ^]^ repurposed a Cu(II)/THPTA complex to act as a catalyst to induce thiazolidine bond cleavage. These catalytic systems allow for the controlled, on‐demand release of a drug payload from bioconjugates under physiological conditions (Figure 4H).^[^ ^109^ ^,^ ^110^ ^]^

## 3 Application in Chemical Biology Research

### 3.1 Biomarker Identification and Diagnostics Imaging Approaches

Biomarkers are biomolecules or processes in the body that are indicative of a disease state. Bioorthogonal chemistry has been used for means of labeling nucleic acids, proteins, and other biomolecules as potential biomarkers. Methods of biomarker identification with bioorthogonal chemistry including bioorthogonal metabolic labeling (BML), post‐translational modification (PTM) enrichment, molecular profiling (omics), and ABPP.

#### 3.1.1 BML

BML relies on the endogenous cellular machinery to integrate bioorthogonal functionality into biomacromolecules allowing for the profiling of dynamic biological processes through the probing. Biomolecules containing the chemical reporter can then be reacted with its complementary bioorthogonal handle allowing for the protein of interest to be detected.

There are two major techniques used in protein labeling: bioorthogonal noncanonical amino acid tagging (BONCAT) and fluorescent noncanonical amino acid tagging (FUNCAT). These techniques are especially useful in proteomic studies for identifying newly synthesized proteins as UAAs are pulse‐fed into a cell to be metabolically incorporated into proteins. In BONCAT, enrichment tags are attached to the incorporated UAA through bioorthogonal chemistry for purification, identification, or quantification purposes, while in FUNCAT, a fluorescent group is added bioorthogonally for fluorescence imaging. From a clinical standpoint, both methods leverage chemical‐reporter‐modified amino acids to identify newly synthesized proteins that are indicative of disease states in clinical samples.^[^ ^15^ ^,^ ^28^ ^,^ ^111^ ^]^

The first example of BONCAT was reported by Dietrich^[^ ^112^ ^]^ to selectively identify, purify, and detect newly synthesized proteins in mammalian cells. Corbin et al.^[^ ^113^ ^]^ used BONCAT to identify proteins prevalent in fragile X (FX) syndrome, a genetic disease that disrupts the FMR1 gene expression leading to adverse brain function. Peripheral blood mononuclear cells were isolated from FX patients and healthy human controls. BONCAT was used to biotinylate nascent proteins which were then isolated with streptavidin beads for further study. Various up‐ or downregulated proteins were identified in FX patients with eleven being considered as potential biomarkers of FX disease identification.^[^ ^113^ ^]^ BONCAT has additionally been shown to work in a variety of in vivo mouse models for similar purposes of identifying proteins associated with various disease states.^[^ ^114^ ^,^ ^115^ ^]^

Furthermore, Taguer et al.^[^ ^116^ ^]^ developed a methodology that uses BONCAT to study the human gut microbiome which has shown significant correlation to cancer and other disease states. BONCAT specific to homopropargylglycine (HPG) in gut microbiota was used in combination with fluorescently activated cell sorting and sequencing to pinpoint translational activity. Human fecal samples incubated with HPG were treated with the Click‐it buffer kit and Alex‐647 azide, which fluorescently tagged the proteins. This method allowed for the examination of translationally active intestinal bacteria.^[^ ^116^ ^]^

FUNCAT adapts UAA tagging to support selective fluorescent imaging within the proteome.^[^ ^117^ ^]^ FUNCAT has been used in vivo mouse models to decipher the proteome of critical limb ischemia. Methionyl‐tRNA synthetase was overexpressed in mesenchymal stem cells to which azidonorleucine was added to replace methionine in protein synthesis. Cells were then subject to bioorthogonal click‐iT reactions with alkyne‐Alexa Fluor 488 allowing proteome wide identification of nascent protein synthesis in critical limb ischemia.^[^ ^118^ ^]^

Livingstone et al.^[^ ^119^ ^]^ used FUNCAT to visualize newly synthesized proteins in neurons. They incubated neurons with HPG, which was subsequently incorporated into newly synthesized proteins. These proteins were then labeled with a fluorescent dye via tetrazine ligation, allowing real‐time tracking of protein synthesis. Using FUNCAT, they demonstrated that secreted amyloid precursor protein‐alpha (sAPPα) significantly increased the synthesis of proteins, specifically Ca^2+^‐permeable AMPA receptors, which are critical for synaptic plasticity. FUNCAT was used to support their conclusion that sAPPα enhances long‐term potentiation by promoting the synthesis and trafficking of these receptors to the synaptic membrane, thereby strengthening synaptic connections and facilitating memory formation.^[^ ^119^ ^]^

The applications of BONCAT and FUNCAT span diverse fields of clinical applications, including neurobiology, disease research, and preliminary protein targeting. Additionally, these methods have been employed to explore developmental biology and tissue‐specific protein dynamics in vivo. As bioorthogonal chemistry continues to evolve, these techniques will undoubtedly remain at the forefront of proteomic research specifically in the field of disease identification.^[^ ^15^ ^,^ ^120^ ^]^

**Figure 5:**
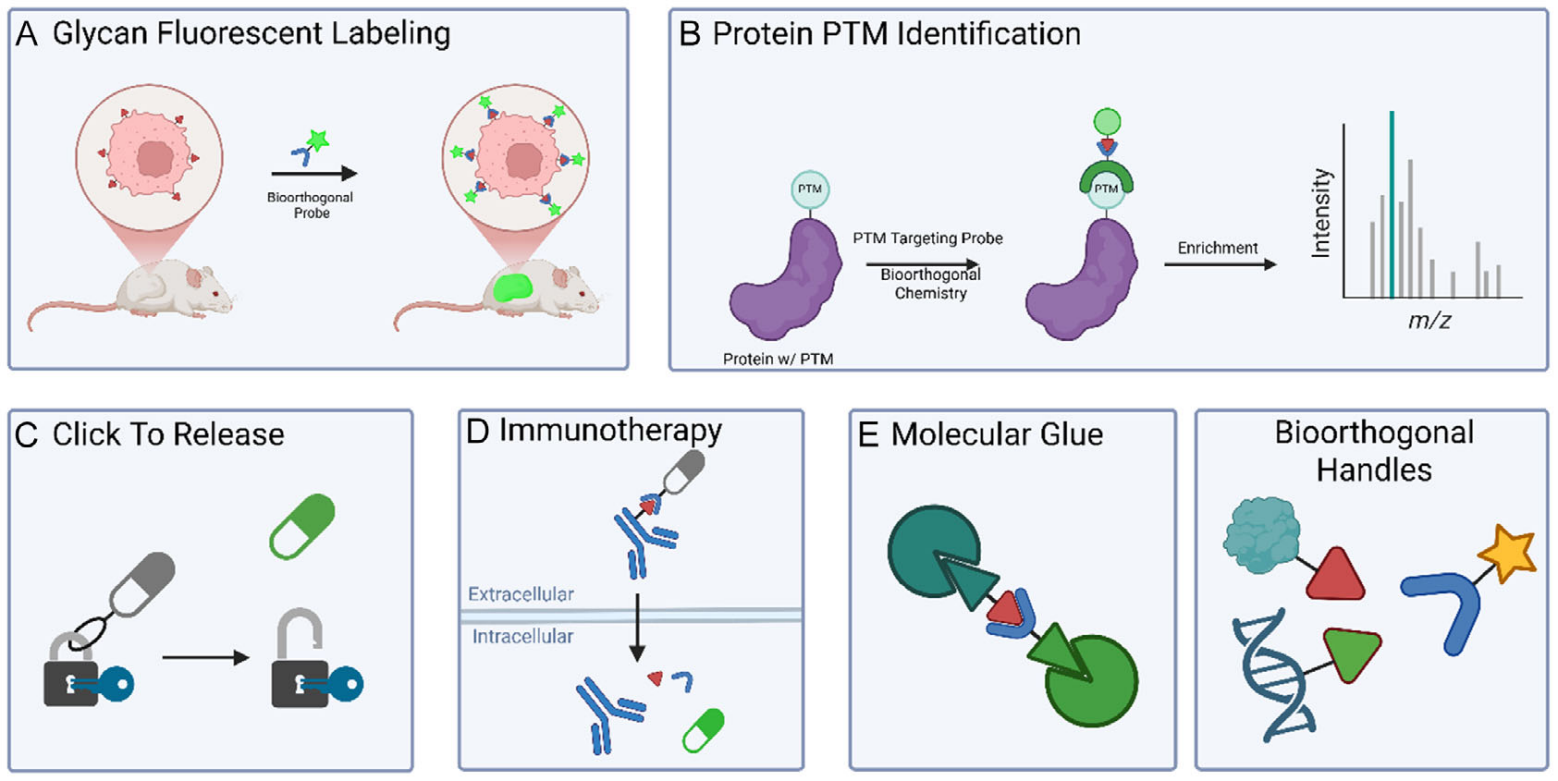
Applications of biocompatible chemistry. A) Metabolic glycan labeling used for cell surface fluorescent enrichment. B) Bioorthogonal PTM labeling that targets endogenous protein PTMs with a bioorthogonal probe for enrichment. C) CTR prodrug releases the active drug molecule from a bioorthogonal scaffold. D) Development of ADC allowing for the targeted delivery of a drug to disease‐specific cells. E) Construction of a two‐headed molecule as molecular glue and labeling targeted biomacromolecules using bioorthogonal handle‐containing building blocks.

Abnormal glycosylation patterns of cell surface proteins have been associated with a variety of disease states, specifically cancer, hence metabolic glycan labeling allows for detection and analysis of these patterns. Bioorthogonal chemistry has been used extensively in the field of metabolic glycan engineering (**Figure** **5**A). Early uses of bioorthogonal chemistry introduced by Bertozzi et al.^[^ ^5^ ^,^ ^121^ ^]^ focused on the manipulation of glycans to study living systems. Azide functionalized synthetic analogs of natural sugars were metabolically incorporated followed by Staudinger ligation allowing for selective labeling of cell surface glycoconjugates.^[^ ^5^ ^,^ ^121^ ^]^ More recently, Leeper et al.^[^ ^122^ ^]^ used methycyclopropene‐tagged acetylated monosaccharides to label cancer cells leveraging the high reaction rate and selectivity of IEDDA reactivity. They found that, in contrast to azide tagged monosaccharides, deacetylation of the monosaccharide enhanced cell labeling offering significant potential for imaging.

Mooney et al.^[^ ^123^ ^]^ reported a strategy for BML of dendritic cells with azido‐modified sugars to modulate their function and enhance immune responses. Immunomodulatory molecules, such as cytokines or antigens, were then attached directly to the cell surface using SPAAC. Though similar to previous approaches this demonstrates targeted modulation and enhanced activation of dendritic cells, antigen presentation, and T cell priming, all critical regulatory processes for effective immune responses. SPAAC enabled precise control over dendritic cells function, offering potential improvements in the efficacy of vaccines and other immunotherapeutic interventions.^[^ ^123^ ^]^

In recent years, Bertozzi et al.^[^ ^124^ ^]^ has utilized BML to study phenolic glycolipids (PGLs) in mycobacteria. PGLs have been shown to be key virulence factors in pathogenic mycobacteria playing a key role in modulating host immune system. BML strategies were employed using azide‐modified phenolic precursors that were metabolically incorporated into PGLs during bacterial growth. Modified PGLs were subsequently tagged and visualized using SPAAC enabling for tracking of localization patterns and function in mycobacterial infections. The study underlines the role in host–pathogen interactions and immune evasion that could play a critical role in the link between cancer and the gut microbiome.^[^ ^124^ ^]^

#### 3.1.2 PTM Biomarker Identification

PTMs significantly enhance proteome diversity and functionality by altering protein structure and function through a variety of biochemical mechanisms. Overall, the addition and removal of PTMs to proteins play a critical regulatory role in protein interactions and enzymatic activity as a means of blocking and/or activating key cellular signaling pathways. Intrinsically, the regulatory nature of PTMs has made the proteins that modify crucial biomarkers in diseases. Protein PTMs are added, recognized, and removed biologically by ‘writer’, ‘reader’, and ‘eraser’ enzymes, respectively, hence, biomarker identification relative to PTMs consists of the protein by which a PTM is added and the enzymes that mediate PTMs.^[^ ^125^ ^]^ Bioorthogonal chemistry has streamlined the process to which protein PTM biomarkers are identified allowing for significant advancements in targeted therapeutic approaches. The most common methods of PTM biomarker identification are mimic based PTM chemical probes, bioorthogonal PTM labeling, and reader bait (Figure 5B).

The basis of PTM chemical probe mimics is to take advantage of the promiscuity of writer enzymes using a substrate analog containing a chemical tag that allows for bioorthogonal labeling and enrichment. For example, bioorthogonal chemical probes were used to characterization serotonylation by transglutaminase 2 (TGM2), a PTM involving the attachment of serotonin to proteins. Bioorthogonal labeling and enrichment techniques to selectively tag and identify serotonylated proteins within the proteome, enable a detailed analysis of this modification in biological systems.^[^ ^126^ ^]^ Additionally, protein methylation profiling was achieved by using S‐adenosyl‐L‐methione (SAM) analogs containing terminal alkyne functionality allowing for selective identification of methylated proteins within the proteome.^[^ ^127^ ^]^ Our lab recently reported the use of a serotonin based bioorthogonal chemical probe to identify TGM2 as the primary writer, eraser, and exchanger enzyme for histone H3 monoaminylation. TGM2 was found to play a critical role in regulating monoaminylation of histones, an essential process in neuronal function and behavior, hence, linking epigenetic regulation to neural activity through monoamine neurotransmitters.^[^ ^128^ ^]^

Additionally, not all protein PTMs require writer enzymes with some being added spontaneously through nonenzymatic covalent modification (NECM). In these cases, chemical probes can be easily added to PTM sites without regard to the promiscuity of the writer enzyme. David et al. utilized alkynyl methylglyoxal probes to investigate NECM of histone glycation reporting the identification of numerous chromatin remodelers, nucleotide binder, and DNA repair proteins that could have significant implication in chromatin biology.^[^ ^129^ ^,^ ^130^ ^]^

Whiles mimic based chemical probes have proven to be extremely effective in PTM biomarker identification, they face the main limitation of competition with endogenous metabolites making them relatively impractical for in vivo applications. Bioorthogonal PTM labeling addresses this concern by targeting the endogenous PTM to characterize proteins of interest. Building off our previous work with TGM2,^[^ ^128^ ^]^ our lab reported the use of bioorthogonal probes to study the regulatory function of histone monoaminylation (i.e. serotonylation and dopaminylation) in cancer cell chromatin. Alkyne function CRACR and strain‐promoted oxidation‐controlled cyclooctyne‐1,2‐quinone cycloaddition‐based probes were used to specifically target serotonylation and dopaminylation respectively on histone H3 to which reporters were attached to the probe via CuAAC. This strategy found that histone monoaminylation is highly enriched in cancer cells due to an overexpression of TGM2.^[^ ^131^ ^]^ Most recently, our lab has also reported the labeling of histone serotonylation using a pH‐controlled regioselective rapid ene‐type reaction (RRER).^[^ ^132^ ^]^ Utilizing alkynyl TAD‐based probes, serotonylated H3Q5 peptide mimic could be successfully labeled, expanding our understanding of the selectivity of TAD‐mediated RRER. As stated previously, a limitation of this method is the nonselective labeling of tryptophan, tyrosine, and serotonin. However, the lack of tryptophan present on histones and pH‐dependent tyrosine selectivity makes this method an effective expansion of the chemical biology toolbox for studying histone monoaminylation, specifically serotonylation.^[^ ^132^ ^]^ Similar strategies of bioorthogonal PTM labeling have also been used to profile glycation, methylation, and citrullination patterns, PTMs that have been shown to have direct effects on numerous disease states.^[^ ^133^, ^134^, ^135^ ^–^ ^136^ ^]^ Limitation of this method, however, resides in the necessity for a highly specific PTM targeting probe which has given this method its own impracticalities for in vivo applications.

The final method used for PTM biomarker identification utilizes ‘bait’ peptides containing the target PTM, an unnatural amino acid with a photocrosslinking group (typically a diazirine), and a bioorthogonal tag to be pulled down. When a reader interacts with the bait PTM, it can be covalently crosslinked and subsequently pulled down after bioorthogonal enrichment.^[^ ^125^ ^]^ One of the most notable applications of this method utilized photolysine and photoarginine bait peptides in order to identify PTM readers on histones for acetylation, methylation, and ubiquitination (only for photo‐lys).^[^ ^137^ ^,^ ^138^ ^]^ These methods subsequently led to the identification of potential reader enzymes including MutS protein homolog 6, repelDouble PHD fingers 2, and Retinoblastoma binding protein 4/7.^[^ ^139^ ^]^ A similar study using photolys bait peptides identified Menin, a protein associated with the MEN1 tumor suppressor gene, to function as a reader of the dimethylated K79 on histone H3. They further demonstrated that Menin specifically recognizes K79me2 within the nucleosome, providing insights into its regulatory role in chromatin and potential implications in cancer. The discovery highlights the importance of Menin in interpreting histone modifications and its involvement in epigenetic signaling pathways.^[^ ^139^ ^]^

#### 3.1.3 Advancements in Molecular Omics Profiling and High‐Throughput Screening (HTS)

Biocompatible chemistry has had extensive implications in the fields of genomics and transcriptomics to study the subtle complexities of nucleic acids. Wu et al.^[^ ^140^ ^]^ used IEDDA for detection of microRNA (miRNA), leveraging rapid and specific reactivity of tetrazines with TCO to improve the sensitivity and efficiency of miRNA detection. An internally quenched tetrazine‐fluorophore compound can react with an azabenzonorbornadiene derivative to amplify the fluorescent signal enabling real‐time monitoring and amplification of the miRNA. The dienophile was attached to either the 5′ or 3′ ends of oligonucleotide probes, hence, modifying probes hybridization with target miRNA triggered the fluorogenic reaction. miR‐21 was particularly targeted as this human miRNA is linked to various cancers, highlighting the potential for sensitive and specific detection of disease‐associated nucleic acids using bioorthogonal means.^[^ ^140^ ^]^ Wu et al. would additionally use similar methods for detecting superfolder GFP mRNA expression. A caged vinyl ether fluorophore would only fluoresce after a proximity‐based reaction with dipyridyl tetrazine allowing live imaging of mRNA expression.^[^ ^141^ ^]^

The use of biocompatible chemistry in reference to nucleic acids has most notably been used to study DNA synthesis and RNA transcription.^[^ ^142^ ^]^ Salic and Mitchison^[^ ^143^ ^]^ reported the metabolic incorporation of an alkyne modified uridine residue, 5‐ethynyl‐2′‐deoxyuridine (EdU), that could be incorporated into DNA during replication and detected using CuAAC. This acted as an early alternative to the traditional method of studying newly synthesized DNA which incorporated 5‐bromo‐2′‐deoxyuridine (BrdU) into DNA followed by antibody‐based detection.^[^ ^142^ ^–^ ^144^ ^]^

Bioorthogonal chemistry has fostered significant advancement in glycomic identification of biomarkers in specific disease states. Bertozzi et al.^[^ ^145^ ^]^ used glycosylation to study the PTM of RNA using azide functionalized sugars to glycosylate RNA followed by CuAAC enrichment with biotin probes allowing metabolic labeling of RNA. Live cell imagining using these methods reported that most glycosylated RNA is localized to the cell surface, expanding the role of RNA in extracellular biology.^[^ ^142^ ^,^ ^145^ ^]^ Similar reports from Kooyk et al.^[^ ^146^ ^]^ would characterize glycosylation‐related genes and pathways in cancer identifying potential cancer‐related biomarkers.

Additionally, Bertozzi et al.^[^ ^147^ ^]^ introduced a glycoproteomic method of studying glycoprotein expression in human prostate cancer tissue that relies on bioorthogonal labeling. Prostate tissue cultures were metabolically labeled with azido‐modified sugars to tag glycoproteins which were subsequently conjugated to probes via CuAAC. Identification through glycoproteomic analysis allowed for the detailed characterization of glycosylation patterns in prostate cancer tissue revealing potential biomarkers and therapeutic targets highlighting the role of glycosylation in cancer biology.^[^ ^147^ ^]^

Biocompatible chemistry has also provided significant advancements in lipidomics, especially in finding protein biomarkers. Reports from de Kroon et al.^[^ ^148^ ^]^ used dual‐functionalized lipid analogs to combine photocrosslinking and click chemistry to specifically identify proteins that interact with phospholipids at the cell membrane. Photoactivatable phospholipid analogs that, upon UV irradiation, covalent bind proteins that transiently interact with the lipid and bioorthogonal handles allowed for biotin enrichment of the lipid–protein complex.^[^ ^148^ ^]^ Similarly, Tafesse et al.^[^ ^149^ ^]^ reported the multifunctional flash and click method to investigate viral infections. Lipid analogs equipped with both photoactivatable and click‐compatible functionality, enabled the precise labeling of lipid–protein interactions during viral entry and replication. Flash and click methods allow the study of viral component interaction with host cell membranes, a critical component for understanding viral infection mechanisms.^[^ ^149^ ^]^

Proteomic biomarker identification has seen the largest growth with advancements in the field of biocompatible chemistry. Building off our previous work in protein monoaminylation biomarker identification,^[^ ^128^ ^,^ ^131^ ^]^ our lab utilized bioorthogonal chemical proteomics to identify the functional roles of serotonylation^[^ ^150^ ^]^ and dopaminylation^[^ ^151^ ^]^ in cancer and offer potential insight into therapeutic intervention. The utilization of bioorthogonal PTM targeting approaches in proteomic studies supports the regulatory roles of TGM2 in cancer development suggesting that targeting the pathways by which TGM2 mediates monoaminylation could serve as a promising anticancer strategy.^[^ ^150^ ^,^ ^151^ ^]^

Bioorthogonal proteomics revolutionized ABPP. The earliest example of what could arguably be considered ABPP dates back to the 1960s, where Strominger et al.^[^ ^152^, ^153^ ^–^ ^154^ ^]^ radioactively labeled penicillin to determine that penicillin binds transpeptidase enzymes later leading to the extensive application of penicillin as a drug. Cravatt would coin the term ABPP in 1999 using active site targeting probes containing a fluorescent reporter or biotin enrichment tag to study serine hydrolases at a proteome wide scale.^[^ ^155^ ^]^ While ABPP advanced protein biomarker identification at a proteome scale, it struggled from one inherent flaw, bulky reporter tags could interfere with the transient binding of small molecule probes to a proteins active site. Biocompatible chemistry offered the significant advantage of a small functionality that allowed for reporter tags to be clicked to the small molecule probe after target interaction has already occurred.^[^ ^156^, ^157^, ^158^ ^–^ ^159^ ^]^ Click‐based ABPP also directly led to a broader mapping of proteins containing a cysteine‐based active site using electrophilic warheads to quantitatively profile the reactivity of cysteine residues across the proteome. This enabled the identification of functional cysteines that play critical roles in protein function and regulation. Heightened nucleophilic reactive profiles of cysteine are often associated with active or allosteric regulatory sites within a protein allowing for a broader study of cysteine‐mediated biochemical processes and the development of targeted covalent inhibitors.^[^ ^160^ ^]^

More recently, ABPP has been used to reveal off‐target inhibition of fatty acid amide hydrolase (FAAH) inhibitors. Recent phase 1 trials for the FAAH inhibitor BIA 10–2474 indicated that it to have severe, adverse neurotoxic effects. Chemoproteomic probes containing a BIA 10–2474 warhead and CuAAC compatible fluorescent reporter were used to systematically analyze its protein interactions across the proteome. Results revealed that BIA 10–2474 also inhibits several other serine hydrolases, including enzymes involved in lipid metabolism and brain function providing a mechanistic explanation for the clinical toxicity of BIA 10–2474. The results of this study revealed the lack of specificity of BIA 10–2474 FAAH inhibitor and resulted in it failing in phase 1 clinical trials.^[^ ^161^ ^]^

Due to the electrophilic nature of SuFEx hubs, it has rather been used as an ABPP warhead. The Van der Hoorn group was among the first to utilize sulfonyl fluorides as probes for ABPP to characterize serine proteases.^[^ ^162^ ^]^ The unique reactivity and stability profile of sulfonyl fluorides made ideal for covalent modification of active serine residues in proteases. SuFEx probes demonstrated selective labeling and identifying serine proteases in complex biological samples, providing insights into protease activity and function. The study acted as a preliminary study for the potential of sulfonyl fluoride ABPP as a versatile tool for studying serine protease, a known biomarker in various biological processes and diseases.^[^ ^162^ ^]^ Kelly and Sharpless^[^ ^163^ ^]^ groups expanded on this work investigating the use of arylfluorosulfates as chemoselective probes targeting tyrosine residues in intracellular lipid‐binding proteins via SuFEx chemistry. Arylfluorosulfates were shown to covalently modify conserved tyrosine residue within lipid binding proteins highlighting the potential of SuFEx to act as modern adaptation of ABPP.^[^ ^163^ ^]^ Further advancements in the specificity of SuFEx chemistry would enhance the use of SuFEx in studying native systems.

#### 3.1.4 Click Chemistry‐based Diagnostic Applications

Advancements in biocompatible chemistry have allowed for significant advancements in diagnostic imaging approaches. IEDDA has been extensively used in the field of clinical radiochemical imaging, leveraging the high reactions kinetics of IEDDA to improve tumor targeting and reduce background noise. Specifically, Lewis et al. has made significant strides in positron emission tomography (PET) imaging which utilizes radioactive tracers to image tissue. Using a human epidermal growth factor receptor 2 (HER2) breast cancer model, Lewis et al.^[^ ^164^ ^]^ covalently coupled a strained norbornene (dienophile) to a well characterized anti‐HER2 antibody trastuzumab followed by treatment with tetrazine substrates bearing radioisotopes ^64^Cu and ^89^Zr. Results proved to be effective in selectively imagining breast cancer in mice models.^[^ ^164^ ^]^ The same group expanded on this approach using a TCO‐modified A33 antibody to selectively target SW1222 colorectal cancer cells with ^64^Cu radioisotopes.^[^ ^165^ ^]^ Similarly, Fox,^[^ ^166^ ^]^ Weissleder,^[^ ^167^ ^]^ and Lewis^[^ ^168^ ^]^ all paired the rapid and selective nature of IEDDA with ^18^F‐labeled probes, expanding the library of PET imaging methods. Liang et al.^[^ ^169^ ^]^ expanded on this work using an IEDDA‐based PET imaging strategy in mice models to monitor PD‐L1 expression in tumors, offering a powerful tool for assessing immune checkpoint status in cancer. While this approach is currently limited to animal models, expansion of this methodology could be broadly used in a variety of clinical applications.

Many of the previously mentioned biocompatible methodologies used for diagnostic imaging have been adapted for more widespread biomedical and clinical applications in the form of click chemistry‐based diagnostic kits with the most notable series being the Click‐iT kits from Fischer Scientific. The primary click‐based reactions used for these kits include CuAAC and SPAAC for their commercial availability and reliability. These labeling kits allow for the detection of biomolecules across a variety of applications while taking advantage of previously mentioned methodologies. In the clinical space, these kits leverage the rapid kinetic of click‐type reactivity with metabolic labeling, PTM detection, and early‐stage protein and nucleic acid synthesis to characterize key biomarkers and of a variety of disease states (**Table** **2**). While the current scope of clinically relevant diagnostic kits requires patient samples to be analyzed in vitro, the nature of biocompatible chemistry expands to allow for real time in vivo diagnostics in the near future.

**Table 2:** Representative commercial Click‐iT kits that leverage biocompatible chemistry for biomarker and diagnostic analyses.

Kit name	Reaction	Target	Applications	Detection method
Click‐iT EdU	CuAAC	DNA	Cell proliferation	Fluorescence
Click‐iT Plus EdU	SPAAC	DNA	Cell viability; Proliferation and function	Fluorescence
Click‐iT EU	CuAAC	RNA	Transcriptional regulation and gene expression	Fluorescence
Click‐iT O‐GlcNAc	SPAAC	Glycosylated proteins	PTM detection	Biotin/Fluorescence
Click‐iT HPG	CuAAC	Proteins	Protein analysis	Fluorescence
Click‐iT AHA	CuAAC	Proteins	Protein analysis	Fluorescence/Biotin
Click‐iT TUNEL	CuAAC	Fragmented DNA	Apoptosis and DNA damage	Fluorescence
Click‐iT ManNAz	CuAAC	Glycoproteins	Metabolic glycan labeling	Fluorescence/Biotin
Click‐iT Palmitic Acid	CuAAC	Lipids	Metabolic glycan labeling	Fluorescence/Biotin

### 3.2 Rapid Construction and Screening of Compound Libraries

Another field that has seen significant advancement is the construction of small molecule drug libraries. HTS methods offer the advantage of identifying potential drug candidates by screening large chemical libraries against diverse biological targets. While recent drug discovery efforts have focused on enzyme families, only a limited number of molecular biomarkers (324 to date) have FDA approved drugs, highlighting the challenges in small molecules drug targeting. Advancements in biocompatible click chemistry methods have revolutionized drug lead discovery and optimization. Small‐molecule libraries created using biocompatible click methods have been instrumental in the development of inhibitors and activity profiles of key enzymes to be optimized as therapeutic targets.^[^ ^170^ ^]^

Click‐type reactions offer the advantage of rapid and highly selective reactivity in small molecule synthesis, reducing the need for extensive purification methods. Chan et al.^[^ ^171^ ^]^ discovered that flavonoid dimers can reverse drug resistance mediated by multidrug resistant protein 1 (MRP1/ABCC1) in cancers. By leveraging the advantages of CuAAC synthesis, a diverse 300‐member library of MRP1 targeting flavonoid dimers were synthesized without need for purification and screened for their ability to inhibit MRP1. This allowed for the subsequent identification of potent compounds that restore chemosensitivity in resistant cancer cells.^[^ ^171^ ^]^

Furthermore, Miyata et al.^[^ ^172^ ^]^ utilized CuAAC to develop a 120‐member library of small molecule inhibitors that target HDAC8, a member of the Type I HDAC family whose overexpression has implications in diseases such as cancer, inflammation, and neurodegenerative disorders. Screening of the initial 120‐member library identified an effective triazole scaffold by which further optimization developed a 31‐member triazole library. This led to the discovery of two small molecules which exhibited significantly higher inhibitory activity against HDAC8 compared to the initial hits.^[^ ^172^ ^]^ The same group would further report the development of a 504‐member library of HDAC3 inhibitors through similar methods that would identify two additional potent and selective HDAC3 inhibitors.^[^ ^173^ ^]^

The para‐quinonoid scaffold was shown to have inhibitory efficacy against cell division cycle 25 (Cdc25) protein phosphatases which have been widely associated with a variety of different cancers. Based on this scaffold Zhan et al.^[^ ^174^ ^,^ ^175^ ^]^ developed a 96‐member library of Cdc25 phosphatase inhibitors using miniaturized click chemistry methods by which synthesis occurs at small scales in a 96‐well plate. They further discovered several compounds with significant inhibitory activity and selectivity for Cdc25 phosphatases.^[^ ^174^ ^,^ ^175^ ^]^ The same group used this method to develop a library of small molecule compounds that target the main protease (M^pro^) of SARS‐CoV‐2 finding several hit compounds that displayed antiviral activity.^[^ ^176^ ^]^

While most click‐based small molecule library constructions utilize CuAAC due to its small functionality and highly selective nature making purification obsolete, SuFEx methods have emerged as a promising method of library construction. A protocol that utilizes SuFExable hubs to develop modular synthesis of functional libraries was recently reported by Moses et al.^[^ ^177^ ^]^ While a specific biological target was not explicitly indicated, it shows that SuFEx offers the potential for rapid and efficient library construction that would not require the triazole scaffold.^[^ ^177^ ^]^ Overall, advancements in click chemistry have enabled the rapid construction of small molecule libraries and subsequent screening for lead hit compounds, accelerating the discovery of new therapeutic agents for a variety of diseases.

### 3.3 Targeted Drug Delivery and Drug Discovery

#### 3.3.1 Click‐to‐Release (CTR) Drug Delivery

Localization and timing of drug administration are two critical components of drug delivery by which adverse effects can result from a drug acting at the wrong place or time. As a result, improvements in drug delivery and release mechanisms have been a key research avenue to improve drug efficacy, control, and minimize potential side effects. Advancements in biocompatible chemistry have had a positive impact on this field as it allows for the selective activation of an inactive form of a drug (prodrug) using an emerging method known as CTR (Figure 5C).^[^ ^15^ ^]^

Early examples of CTR prodrug activation methods reported by Florent^[^ ^178^ ^]^ and Robillard^[^ ^179^ ^]^ reported Staudinger ligation mediated release of doxorubicin, a potent anticancer therapeutic, in cell culture and in vivo applications. While these examples served as proof of concept for biocompatible CTR drug delivery, Staudinger ligation's sluggish kinetics made it difficult to control the drug concentration at the specific site of action.^[^ ^178^ ^,^ ^179^ ^]^ Gamble et al.^[^ ^180^ ^]^ reported selective prodrug activation through methods of SPAAC using TCO to initiate the release of doxorubicin. While this method shows complete release of the payload and further addressed kinetic issues faced by Staudinger ligation CTR methods, the SPAAC mediated prodrug faced stability issue limiting its in vivo applications.^[^ ^180^ ^]^

CTR methods of prodrug activation hit stride in methods involving IEDDA using TCO and tetrazine due to its rapid reaction kinetics. Collaborative efforts from Oneto and Royzen^[^ ^181^ ^,^ ^182^ ^]^ have recently made significant progress using tetrazine‐TCO‐based CTR methods for product activation and delivery. The drug is attached to the TCO moiety by an allylic ether, carbamate, or ester allowing for an induced release upon tetrazine ligation. Biopolymer with biocompatible functionality induced localization and activation of the prodrug resulting in high local concentration within cells. Through β‐elimination of the carbamate, carbonate, or ether, the drug is freed and taken up by tumor cells resulting in selective drug targeting. This method of CTR prodrug delivery was presented using doxorubicin resulting in an 80‐fold decrease of toxicity in mice models relative to standard doxorubicin treatments.^[^ ^181^ ^,^ ^182^ ^]^ Additionally, this method has been taken to phase I clinical trials by Shasqi, a click chemistry focused biotech company based out of California, as the first clinical study of a click chemistry‐activated prodrug.^[^ ^183^ ^]^ Additionally, Bernardes et al.^[^ ^184^ ^]^ also reported traceless release of duocarmycin, a potent anticancer drug, using IEDDA‐based CTR. The alcohol group on duocarmycin is protected by a vinyl ether to which the reaction with tetrazine releases the active form of duocarmycin in live cells to induce cytotoxic effects.^[^ ^183^ ^]^

Taran et al.^[^ ^185^ ^]^ reported CTR methods using sydnonimine and cycloalkynes as proof of principle for SPSIC based drug CTR technologies. The same group would advance this methodology to perform drug synthesis of sorafenib, an anticancer drug, in cells through bioorthogonal activation in micelle nanoreactors. Micelle amphiphiles were constructed with half of the sorafenib structure (split at the urea scaffold) linked to the PEG head through a sydnonimine scaffold. Sydnonimine then reacts with cyclooctyne through SPSIC, releasing the half‐sorafenib as a reactive isocyanate subsequently reacting with the primary amine on the complementary half of sorafenib to produce the active form of sorafenib. In vitro studies displayed strong antiproliferative and proapoptotic effects in HepG2 cells expanding the potential of in situ synthetic methods of drug delivery.^[^ ^186^ ^]^

#### 3.3.2 Immunotherapy and Antibody‐Based Therapeutics

Antibody drug conjugates (ADCs) have recently emerged as a promising means of drug delivery using a monoclonal antibody for drug localization. ADCs are composed of cytotoxic drug covalently linked to a monoclonal antibody leveraging high specificity targeting approached and high potency therapeutics (Figure 5D). The first class of therapeutically approved ADCs utilized Gemtuzumab ozogamicin, a monoclonal antibody that targets the CD33 surface protein on acute myeloid leukemia cells to deliver calicheamicin, a cytotoxic antibiotic.^[^ ^187^ ^]^ Among the 14 FDA approved ADC drugs, there are two common methods of payload delivery: hydrazone and disulfide cleavage. Hydrazone based linkers rely on pH dependent hydrolysis of a hydrazone linkage to initiate payload delivery. The linker is stable in blood circulation but triggers cleavage in slightly acid environments of the lysosome (pH 4.8) and endosome (pH 5.5–6.2) of cancer cells.^[^ ^187^ ^,^ ^188^ ^]^ Disulfide linkers, on the contrary, release the payload upon disulfide cleavage initiated by glutathione (GSH). Disulfide based ADCs are stable in blood circulation due to the low concentration of GSH in the blood and a high intracellular concentration in cancer cells as GSH plays a critical role in cell survival, proliferation, and differentiation. Additionally, linkers that undergo proteolytic activation have also received FDA approval.^[^ ^188^ ^]^ Endogenous mechanisms of linker cleavage have shown premature payload release underlining the need for alternatives that utilize inducible ADC activation.

Much of the literature regarding biocompatible ADCs has focused on controlling the concentration of a drug release by ADCs through different drug‐to‐antibody ratios. Building off the work of GlycoConnect, van Berkel et al.^[^ ^189^ ^]^ used glycan engineering to selectively install azide sites on trastuzumab, a monoclonal antibody that targets HER2 which is overexpressed in breast cancer cells. SPAAC is then performed using BCN derivatives that contain a proteolysis activated prodrug. This method allows single payload release of monomethyl‐auristatin E (MMAE), a potent antimitotic agent, using ADCs to control local drug concentrations. Additionally, it has been shown to be compatible with other cytotoxic payloads.^[^ ^175^ ^,^ ^176^ ^]^ While this method shows good efficacy as a new generation of ADCs, it is limited by the need for complex antibodies and difficult purification strategies.^[^ ^189^, ^190^, ^191^ ^–^ ^192^ ^]^

Biocompatible CTR methods have recently revolutionized methods of ADC drug delivery methods. Robillard et al.^[^ ^193^ ^]^ utilized IEDDA induced CTR by using an antitumor associated glycoprotein‐72 (Anti‐TAG72) diabody which was TCO‐linked to MMAE. Anti‐TAG72 targets glycoproteins on tumor cells surfaces taking advantage of the upregulation of protein glycosylation in tumor cells. Tetrazine treatment subsequently activates the release of MMAE for bioorthogonal ADC induced targeted therapy. This method proved to have strong anticancer activity in vitro and in vivo in mice with colorectal and ovarian cancer leading to survival without signs of toxicity for 4 months. Additionally, the FDA approved ADC Adcetris which also used MMAE as the payload except with a protease‐cleavable linker was used with the same anti‐TAG72 showing no effects in tumor models. This exacerbates the need for nonendogenous alternatives to ADC activation.^[^ ^193^ ^]^

Bioorthogonal chemistry has also been employed into other realms of antibody‐based therapeutics, most notably CAR‐T cells. Lian et al.^[^ ^194^ ^]^ developed CAR‐T cells bioorthogonally equipped with hyaluronidase (HAase) and the checkpoint blocking antibody α‐PDL1. This was accomplished through the metabolic incorporation of azides to the cell surface and subsequent attachment of HAase and α‐PDL1 through bioorthogonal click chemistry. HAase acts to degrade hyaluronic acid with the extracellular matrix of tumor microenvironments allowing for CAR‐T cells to penetrate deeper into solid tumors. In the low pH environment of tumor microenvironments, the DBCO linker that connects α‐PDL1 is cleaved, allowing it to act as a checkpoint inhibitor.^[^ ^194^ ^]^ The incorporation of bioorthogonal chemistry to antibody‐based therapeutics shows the potential for advancements in technology related to immunotherapies.

#### 3.3.3 Molecular Glue and Targeted Protein Degradation

Naturally, biocompatible chemistry has seen a significant amount of use in the field of molecular glue development as a means of crosslinking and manipulating interactions between biomacromolecules. Notably, click chemistry has extensively been used to develop hetero‐bifunctional molecular glues acting as a promising means of PTM biomarker identification and have recently gained significant stride in introducing a PTM onto a protein of interest (Figure 5E). Jin et al.^[^ ^195^ ^]^ developed a strategy using an acetylation targeting chimera to induce acetylation of the tumor suppressor p53. By taking advantage of the histone acetyltransferase p300/CBP complex, p53 mutants were successfully acetylated as a proof of concept for the potential use of molecular glues as agonists for anticancer signaling pathways.^[^ ^195^ ^]^ Similarly, Choudhary et al.^[^ ^196^ ^]^ developed a phosphorylation‐inducing chimeric small molecule that take advantages of two kinases (AMPK and PKC) to successfully phosphorylate proteins that would not otherwise be phosphorylation substrates of these kinases.

Molecular glues have specifically seen the most advancement in the realm of targeted degradation technologies. Proteolysis‐targeted chimeras (PROTAC) are hetero‐bifunctional molecules containing three components: a protein targeting warhead, an E3 ubiquitin ligase recruiter, and a linker. PROTACs utilize a cell's natural ubiquitin‐proteasome degradation machinery to mediate targeted disposal of proteins. Naturally, click chemistry methods have streamlined synthesis of PROTAC molecules allowing for the rapid advancement of degradation‐based technologies in drug discovery.^[^ ^197^ ^]^

Heightman et al.^[^ ^198^ ^]^ reported the intracellular self‐assembly of a PROTAC called in‐cell self‐assembly PROTAC (CLIPTAC) to induce targeted degradation of bromodomain‐containing protein 4 (BRD4) which is associated with a variety of disease states including cancer. Two complementary fragments with a TCO‐containing warhead and tetrazine‐containing E3 ligand were allowed to independently bind their target proteins, only forming an active PROTAC upon bioorthogonal chemistry within the cell. Advantages of this approach include minimizing premature protein degradation and improvements in pharmacokinetic control.^[^ ^198^ ^]^ Additionally, similar methodologies that use photo‐click methods of intracellular PROTAC assembly and/or decaging (PHOTAC) have been employed to further leverage pharmacokinetic control of inducible protein degradation.^[^ ^199^, ^200^, ^201^, ^202^ ^–^ ^203^ ^]^

Biocompatible click methods have also allowed for the recent innovative advancement of CTR‐based PROTAC delivery. Wang et al.^[^ ^204^ ^]^ used bioorthogonal chemistry to enhance selectivity and reduce off‐target effects of PROTAC based methods. An inactive form of a PROTACs is conjugated to a TCO‐based masking groups that is selectively cleaved upon tetrazine ligation. This method takes inspiration from CTR drug delivery methods as a means of minimized off‐target effects and inducing localized toxicity. The approach demonstrates potent and selective protein degradation of BET proteins and androgen receptors which have both been shown to have significant cancer implications highlighting the potential of CTR PROTACs to improve therapeutic precision in disease treatment.^[^ ^204^ ^]^

Finally, in 2020 Bertozzi et al.^[^ ^205^ ^]^ developed a new hetero‐bifunctional chimera that uses the lysosome to degrade target protein rather than the proteosome calling this method lysosome‐targeting chimeras (LYTAC). Similar to PROTACs, LYTACs utilize a protein targeting warhead and linker; however, in contrast, LYTACs use a lysosome‐targeting antibody instead of an E3 recruiter. Rather than ubiquitination, the antibody in LYTACs allows the protein of interest to be integrated into the lysosome where it is degraded by digestion enzymes. LYTACS also have the advantage of targeting membrane‐bound proteins, which cannot be targeted by PROTACs, and cell permeability is not an issue as they are designed to undergo endocytosis. In some contexts, however, these advantages can also be limitations.^[^ ^205^ ^,^ ^206^ ^]^ While much of the research in LYTACs in reference to click‐chemistry pertains to a click‐based linker, bioorthogonal chemistry has also been used to improved LYTAC efficiency. Liu et al. leveraged bioorthogonal chemistry to covalently conjugated a LYTAC on the cell surface in DNA‐based LYTAC drugs. This strategy allows for increased retention of the LYTAC on the cell membrane increasing the degradation efficiency.^[^ ^207^ ^]^ Additionally, Qu et al. reported a bioorthogonal‐activated LYTAC which leveraged CuAAC to improve local drug concentration and minimized side effects.^[^ ^208^ ^]^

## 4 Summary and Future Perspectives

Since its emergence in the early 2000s, biocompatible chemistry (including click and bioorthogonal chemistry) has emerged as a transformative tool in chemical biology and drug development. The use of highly selective and high yielding chemical reactions that can be used within living systems without interfering with native biochemical processes has served as a powerful tool in modern science. The field emerged from a philosophy that aimed to mimic the chemical reactions that occur endogenously as a means of studying and manipulating biomolecules in their native environments. The versatility of biocompatible chemistry lies in unique reactivity profiles of the toolbox. As extensively highlighted throughout this review, chemical transformations including the Staudinger ligation, copper‐based cycloaddition, strain‐promoted cycloadditions, and tetrazine‐based chemical methods have displayed continued advancement as a means of addressing the limitations of its predecessors.

Looking forward, biocompatible chemistry continues to expand its reach, with additional emerging applications in synthetic biology, materials science, and diagnostics. In the realm of clinical research, bioorthogonal chemistry is still limited by its in situ application, specifically in the realm of diagnostic approaches for applications in a hospital setting. The ability to selectively label tumor cells during surgery using bioorthogonal methods would increase the chances of complete tumor removal. Recent advancements in imaging technologies have greatly expanded in vivo studies in animal research, especially in cancer‐related investigations. Additionally, it has allowed for refinement of established imaging methods including magnetic resonance imaging, PET, and computed tomography to the degree of enhanced miniaturization and higher‐resolution visualization. Currently, no technique exists that can directly provide results of molecular‐level cellular reactions, rather, such reactions require indirect methods of monitoring through signaling mechanisms and fluorescent or bioluminescent visualization.

While challenges remain for such optimistic applications due to clinical scalability, the field's trajectory has recently seen a significant shift in the realm of drug delivery and targeted drug therapies. The literature remains relatively undersaturated in the field of CTR‐based ADCs whose advancement could revolutionize methods of disease specific drug delivery. The growing field of molecular glue‐based approaches using bioorthogonal chemistry remains in its adolescence with a need for innovative therapeutic methods of bioconjugation that extend beyond diagnostics and labeling. Additionally, biocompatible click‐type methods that aim to target endogenous intracellular activity remain elusive. While methods such as SuFEx‐based ABPP and biocompatible PTM labeling have taken center stage in the realm of biomarker identification, they still struggle from selectivity issues that limits them from being truly ‘bioorthogonal’.

Regardless, the development of biocompatible chemistry has seen unprecedented advancement over the past two decades. As the versatility of this plug‐and‐play toolbox continues to expand, its wide‐reaching applications will see similar expansion. While researchers may never be able to truly mimic the biological selectivity offered by enzymatic reactivity, biocompatible chemistry offers a means of mimicking endogenous chemical reactions and a powerful strategy for chemical biology studies, especially biomedical and clinical applications.

## Conflict of Interest

The authors declare no conflict of interest.
